# Soluble E-cadherin-CXCL1-CXCR2 axis as a therapeutic vulnerability in inflammatory breast cancer brain metastasis

**DOI:** 10.1093/neuonc/noag012

**Published:** 2026-01-23

**Authors:** Xiaoding Hu, Yun Xiong, Emilly S Villodre, Huimin Zhang, Isabella R Longa, Juhee Song, Natalie Fowlkes, Savitri Krishnamurthy, Marissa Rylander, Chandra Bartholomeusz, Debu Tripathy, Wendy A Woodward, Junjie Chen, Bisrat G Debeb

**Affiliations:** Section of Translational Breast Cancer Research, Department of Breast Medical Oncology, The University of Texas MD Anderson Cancer Center, Houston; MD Anderson Morgan Welch Inflammatory Breast Cancer Clinic and Research Program, The University of Texas MD Anderson Cancer Center, Houston; Department of Experimental Radiation Oncology, The University of Texas MD Anderson Cancer Center, Houston; Section of Translational Breast Cancer Research, Department of Breast Medical Oncology, The University of Texas MD Anderson Cancer Center, Houston; MD Anderson Morgan Welch Inflammatory Breast Cancer Clinic and Research Program, The University of Texas MD Anderson Cancer Center, Houston; Department of Experimental Radiation Oncology, The University of Texas MD Anderson Cancer Center, Houston; Section of Translational Breast Cancer Research, Department of Breast Medical Oncology, The University of Texas MD Anderson Cancer Center, Houston; MD Anderson Morgan Welch Inflammatory Breast Cancer Clinic and Research Program, The University of Texas MD Anderson Cancer Center, Houston; Department of Biostatistics, The University of Texas MD Anderson Cancer Center, Houston; Department of Veterinary Medicine and Surgery, The University of Texas MD Anderson Cancer Center, Houston; Department of Pathology, The University of Texas MD Anderson Cancer Center, Houston; MD Anderson Morgan Welch Inflammatory Breast Cancer Clinic and Research Program, The University of Texas MD Anderson Cancer Center, Houston; Department of Biomedical Engineering, The University of Texas at Austin, Austin; Section of Translational Breast Cancer Research, Department of Breast Medical Oncology, The University of Texas MD Anderson Cancer Center, Houston; MD Anderson Morgan Welch Inflammatory Breast Cancer Clinic and Research Program, The University of Texas MD Anderson Cancer Center, Houston; Section of Translational Breast Cancer Research, Department of Breast Medical Oncology, The University of Texas MD Anderson Cancer Center, Houston; MD Anderson Morgan Welch Inflammatory Breast Cancer Clinic and Research Program, The University of Texas MD Anderson Cancer Center, Houston; Department of Breast Radiation Oncology, The University of Texas MD Anderson Cancer Center, Houston; MD Anderson Morgan Welch Inflammatory Breast Cancer Clinic and Research Program, The University of Texas MD Anderson Cancer Center, Houston; Department of Experimental Radiation Oncology, The University of Texas MD Anderson Cancer Center, Houston; Section of Translational Breast Cancer Research, Department of Breast Medical Oncology, The University of Texas MD Anderson Cancer Center, Houston; MD Anderson Morgan Welch Inflammatory Breast Cancer Clinic and Research Program, The University of Texas MD Anderson Cancer Center, Houston

**Keywords:** brain metastasis, CXCL1-CXCR2, inflammatory breast cancer (IBC), metastatic IBC, soluble E-cadherin (sEcad)

## Abstract

**Background:**

The brain is a common site of relapse in inflammatory breast cancer (IBC), an E-cadherin-positive, aggressive form of breast cancer. Elevated serum levels of soluble E-cadherin (sEcad), an 80-kDa fragment, correlated with poorer outcomes and increased brain metastases in patients with metastatic IBC. We hypothesize that sEcad is a driver of brain metastasis in IBC.

**Methods:**

Serum sEcad levels from 348 IBC patients were quantified by ELISA. To examine sEcad function, we used recombinant sEcad protein and generated stable IBC cell lines by cloning and overexpressing Flag-tagged sEcad. Control and sEcad-overexpressing MDA-IBC3 and SUM149 cells were injected into SCID/Beige mice to evaluate brain metastasis burden and survival, and a brain-permeable CXCR2 inhibitor was also tested for efficacy in these models.

**Results:**

Higher serum sEcad levels correlated with poorer overall survival, earlier metastasis, and increased brain metastasis. In vitro, recombinant sEcad and stable sEcad overexpression in IBC cell lines promoted invasion, resistance to anoikis, and activation of pro-survival NF-κβ signaling. In vivo, mice injected with sEcad-overexpressing IBC cells had increased metastatic burden and reduced overall and brain metastasis-free survival. Further, sEcad induced reactive astrocytosis through the CXCL1/CXCL8-CXCR2 axis, and treatment with a brain-permeable CXCR2 antagonist reduced metastatic burden and prolonged survival in the brain metastasis models.

**Conclusion:**

sEcad drives brain metastasis by promoting invasion and anoikis resistance in cancer cells and inducing an inflammatory brain microenvironment via a targetable CXCL1/CXCL8-CXCR2 axis. These findings uncover a novel and critical role for sEcad and highlight CXCR2 as a therapeutic target in patients with metastatic IBC.

Key Points

- High serum soluble E-cadherin (sEcad) levels correlate with poor survival and brain metastasis in inflammatory breast cancer (IBC) patients.
- sEcad drives brain metastasis progression in IBC mouse models.
- Treatment with a brain-permeable CXCR2 antagonist reduces metastasis burden and improves survival in IBC brain metastasis mouse models.

Importance of the Study

Brain metastasis is a common and lethal complication of IBC, with no effective therapies and poorly understood mechanisms. In this study, we identify sEcad as a biomarker of increased risk of brain metastasis and poor survival in IBC patients and demonstrate that sEcad drives brain metastasis in preclinical IBC models by inducing a pro-inflammatory brain microenvironment via the CXCL1-CXCR2 axis. Importantly, treatment with a brain-penetrant CXCR2 antagonist significantly reduced metastasis and prolonged survival in mouse models, revealing a novel therapeutic strategy for IBC brain metastases.

Brain metastasis is a common and challenging manifestation of solid malignancies of the lung, skin, and breast, and is diagnosed in about 200 000 patients per year in the United States.^1^ Brain metastasis occurs in 25%-50% of patients with advanced breast cancer, particularly those with the aggressive HER2-positive (HER2+) or triple-negative breast cancer (TNBC) subtypes.^2^ Therapy options for brain metastases are limited and the current treatment portfolio is ineffective, and most patients die within months, underscoring the critical need for new and effective treatments. However, the development of new therapies against brain metastasis is hampered by our limited understanding of the mechanisms that confer the competence of breast cancer cells during the numerous steps of the brain metastasis cascade. These include cancer cell migration and invasion from the site of the primary tumor into the bloodstream, survival within the high-stress circulatory environment, extravasation from blood capillaries into distant organs, and adaptation to the unique microenvironment of the brain.^3^

Brain metastasis is particularly common in IBC,^4^^,^^5^ a rare, highly aggressive form of breast cancer that in the United States accounts for 2%-4% of all breast cancer cases but contributes to up to 10% of breast cancer-related deaths.^6^ All patients with IBC present with lymph node involvement and one-third present with distant metastasis at diagnosis. HER2+ and TNBC subtypes are overrepresented in IBC,^7^^,^^8^ and more than 37% of patients with HER2+ IBC experience brain metastasis as the first site of relapse.^9^ IBC is currently treated with a multimodality approach consisting of systemic chemotherapy, surgery, and radiation therapy; however, the 5-year survival rates remain poor. A better understanding of the unique biology of IBC and identification of the molecular factors driving its aggressive and metastatic phenotype are urgently needed.

The overexpression of E-cadherin is a notable finding that distinguishes IBC from other breast cancers. E-cadherin expression has been reported as an indicator of low metastatic potential in many cancers; indeed, the loss of E-cadherin expression contributes to increased proliferation, invasion, and metastasis in some breast cancer models.^10^^,^^11^ However, recent studies have demonstrated that E-cadherin also has pro-metastatic roles.^12^^,^^13^ In IBC, E-cadherin is overexpressed in tumor cells, tumor emboli, metastases, and IBC cell lines.^14–19^ The presence of E-cadherin augments invasion and tumorigenesis in preclinical in vitro and in vivo IBC models^14^^,^^20–22^ and supports the formation of tumor emboli, a hallmark of IBC.^17^^,^^22^ These findings strongly support an oncogenic role for E-cadherin in IBC and possibly other tumors with high E-cadherin expression, like ovarian cancer, glioma, invasive ductal carcinomas of the breast, gastric cancer, and some subtypes of prostate cancer.^23–32^

E-cadherin is synthesized as a 120-kDa transmembrane glycoprotein; however, it can be cleaved off the ectodomain and released as a soluble form, designated soluble E-cadherin or sEcad. sEcad consists of an 80-kDa proteolytic fragment that is recognized as a biomarker of progression or recurrence in various cancers.^33–36^ It has also shown tumor promoter functions through various mechanisms and activation of signaling pathways.^24^^,^^28^^,^^37–46^ For example, sEcad disrupts epithelial cell–cell adhesion, a major barrier against cancer cell mobility, thereby increasing the migration and invasion of cancer cells.^37^^,^^38^ It promotes disassembly of the E-cadherin–β-catenin adhesion complex and releases β-catenin to potentiate oncogenic Wnt signaling.^38–40^ sEcad also enhances cell survival by inhibiting apoptosis via functional interaction with membrane-bound E-cadherin and activation of epidermal growth factor receptor (EGFR)-mediated PI3K/Akt/mTOR signaling.^24^^,^^41^ Further, sEcad increases the expression of metalloproteinases to promote tumor invasion into the stroma.^38^^,^^42^ Interestingly, sEcad may act as a soluble growth factor ligand that binds receptors such as HER1, HER2, and HER3 to activate EGFR signaling and promote aggressive tumor growth.^43–46^ sEcad can also be released within exosomes to promote tumor angiogenesis via activation of β-catenin and NF-κB signaling.^28^ However, the roles of sEcad in breast cancer brain metastasis and IBC biology remain underexplored.

Here we report a direct correlation between elevated serum sEcad levels and increased development of brain metastasis and reduced survival in patients with metastatic IBC. We further identified sEcad as a key driver of brain metastasis in IBC mouse models. We further discovered that sEcad promotes invasion, anoikis resistance, endothelial cell adhesion, and trans-endothelial migration, and NF-κB signaling activation in tumor cells while inducing reactive astrocytosis in the brain microenvironment through the CXCL1/CXCL8-CXCR2 axis. With these findings, we propose a novel mechanism of brain metastasis in which sEcad functions as both a mediator of apoptotic resistance within tumor cells and a modulator of the brain microenvironment, thereby facilitating tumor cell survival, seeding, and outgrowth in the brain.

## Methods

### Plasmid Construction

Soluble E-cadherin cDNA (1-707aa) was amplified from E-cadherin (Gene ID: 999) cDNA and then cloned into modified LentiV_Blast-Flag (#111887, Addgene).

### Enzyme-Linked Immunosorbent Assay of IBC Samples

Serum from 348 IBC patients was analyzed for sEcad by ELISA (R&D Systems #DCADE0) in duplicate under IRB-approved protocols. Conditioned medium from cancer cells cultured for 36 hours in serum-free medium was assayed for CXCL1, CXCL8, and DKK1 by ELISA (#DGR00B, #D8000C, #DKK100B; details in Supplementary Methods).

### Cell Cultures

SUM149 and SUM190 cells were purchased from Asterand, MDA-IBC3 was generated in Dr Woodward’s lab, and BCX010 was kindly provided by Dr Meric-Bernstam (details in Supplementary Methods).

### Anoikis Assay

The anoikis assay has been described previously.^47^ Briefly, cells (1 × 10⁶/mL) were cultured in poly-HEMA-coated (10 mg/mL; Sigma) plates for 24 hours, followed by FITC-conjugated Annexin V staining and flow cytometry.

### Lentiviral Production and Transduction

Stable Flag-tagged sEcad-overexpressing IBC cell lines (MDA-IBC3, SUM190, SUM149, BCX010) were generated by lentiviral transduction (details in Supplementary Methods).

### Western Blotting, Antibodies, and Reagents

Details in Supplementary Methods.

### In Vitro Migration and Invasion Assays

Protocol details are given elsewhere^14^ and in Supplementary Methods. Migration and invasion of SUM149 or BCX010 Control/sEcad cells (5 × 10^4^) were assessed in transwells. After 24 hours, migrated cells were fixed, stained, and quantified in 10 random fields using ImageJ.

### Soft Agar/Anchorage-Independent Growth Assay

The soft agar assay was used as described elsewhere^47^^,^^48^ (details in Supplementary Methods).

### Immunohistochemical and Immunofluorescence Staining

Details of the immunofluorescence staining protocol for cultured cells are given in Supplementary Methods.

### Proteome Profiler Arrays

Human cytokine arrays (#ARY005B, R&D) were used according to the manufacturer’s instructions. Briefly, MDA-IBC3-Con/sEcad and SUM149-Con/sEcad cells were serum-starved for 24 hours and fresh growth medium was added. . To prepare samples for the cytokine array, 700 μL of cell culture medium pooled from 3 independent replicates was used. Arrays were stained and imaged with the Bio-Rad gel documentation system and quantified with ImageJ.

### Endothelial Cell Adhesion Assay

Human brain microvascular endothelial HBEC-5i cells were cultured in 6-well plates for 24 hours to form confluent monolayers. GFP-labeled sEcad-overexpressing or control MDA-IBC3/SUM149 cells were added for 30 minutes, washed with PBS, and adherent cells imaged and quantified^49^ (details in Supplementary Methods).

### In Vitro Trans-Blood-Brain Barrier Migration Assay

Protocol details are given elsewhere.^49^ Briefly, mouse astrocytes C8-D1A (5 × 10^5^) were plated on the bottom side of a transwell, and cell medium was refreshed every 15 minutes for 6 hours. The transwell was then inverted back and 2.5 × 10^5^ bEnd.3 cells were plated on the top side of the membrane, after which the transwell was incubated at 37°C for 3 days to allow formation of blood-brain barrier.

### In Vitro Angiogenesis Assay

An in vitro angiogenesis kit (Abcam, ab204726) was used for this experiment (details in Supplementary Methods).

### cAMP Assay Analysis

The cAMP Assay kit (Abcam ab234585) was used (details in Supplementary Methods).

### Animal Studies

Animal experiments were done in accordance with protocols approved by the Institutional Animal Care and Use Committee of MD Anderson Cancer Center, and mice were euthanized when they met the institutional euthanasia criteria for tumor size and overall health condition. Animal care and use were in accordance with institutional and NIH guidelines. For in vivo brain metastasis studies, control and sEcad-overexpressing SUM149 cells (2 × 10^5^) were injected intracardially and MDA-IBC3 cells (1 × 10^6^) were injected into the lateral tail veins in 4- to 6-week-old female SCID/Beige mice (*n* = 10 per group) (Harlan Laboratories). In brain metastasis studies, mice received daily CXCR2-IN-1 (2 mg/kg) treatment via oral gavage. The “early treatment” was begun a day before the injection of sEcad cancer cells. The “late treatment” was initiated 4 weeks after cancer cell injection.

### Statistical Analysis

Characteristics of patients with IBC were summarized by serum sEcad level and compared between patients with sEcad-Low (<95 ng/mL) or sEcad-High (>95 ng/mL) levels (99.8 ng/mL was the 90th percentile). Univariate and multivariate Cox regression analyses were used for overall survival and breast cancer-specific survival as outcome variables. Univariate and multivariate Fine–Gray models were used to consider time to metastasis and time to brain metastasis as outcome variables, and death without an event of interest was considered a competing risk event in the Fine–Gray model. The Kaplan–Meier method was used to estimate survival probabilities, and cumulative incidence was estimated by the Aalen–Johansen method. Log-rank tests were used to compare survival curves. The Gray test was used to compare cumulative incidence curves. *P* values of <.05 indicated statistically significant differences. SAS 9.4 (SAS institute Inc) was used for data analysis. All in vitro experiments were repeated at least 3 times, and graphs depict mean ± SEM. Statistical significance was determined with Student’s *t* tests (unpaired, 2-tailed) unless otherwise specified. GraphPad software (GraphPad Prism 8) was used.

## Results

### Serum sEcad Levels Correlate with Increased Development of Brain Metastasis and Poor Clinical Outcomes in Patients with IBC

In previous studies,^50^ we generated 2 sublines from a HER2+ metastatic IBC cell line, which we designated MDA-IBC3.1 (a weakly brain-metastatic subline) and MDA-IBC3.2 (a highly brain-metastatic subline). These sublines demonstrate distinct differences in their ability to metastasize to the brain after tail-vein injection into mice. Given that E-cadherin is consistently overexpressed in IBC tumor cells, tumor emboli, and metastases, and has been shown to have an oncogenic role in IBC,^15–19^ we examined the expression of E-cadherin in lysates from these 2 sublines. Interestingly, we observed remarkably higher expression of sEcad, but not full-length E-cadherin, in the highly brain-metastatic cells relative to the weakly brain-metastatic cells (Supplementary Figure S1A). These findings were confirmed in the cell supernatants by ELISA (Supplementary Figure S1B), thus correlating sEcad to the propensity to form IBC brain metastasis in these sublines.

**Figure 1.**
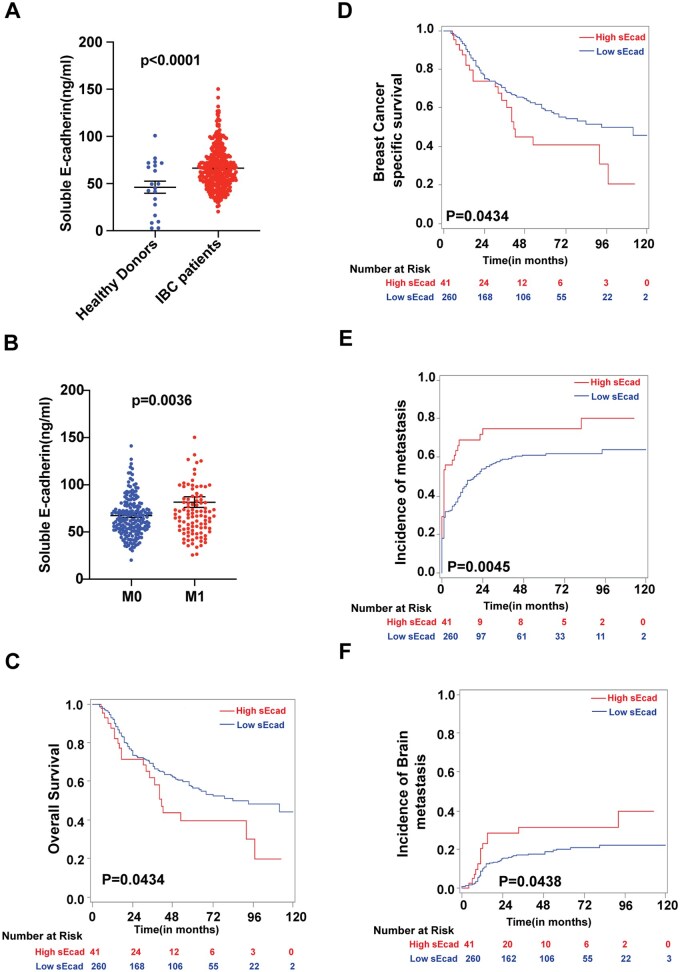
Higher serum soluble E-cadherin (sEcad) levels correlate with increased development of brain metastasis and poor survival outcomes in patients with inflammatory breast cancer (IBC). sEcad serum levels were significantly elevated in IBC patients relative to healthy donor serum samples (*P *< .0001). (B-F) Higher serum sEcad levels were associated with (B) metastasis status, (C) reduced overall survival, (D) reduced breast cancer-specific survival, (E) increased risk of metastasis, and (F) increased risk of brain metastasis.

To examine the clinical relevance and overall significance of sEcad in patients with IBC, we measured serum levels of sEcad by ELISA in samples from 348 patients with IBC accrued to a prospective registry from the dedicated IBC clinic at The University of Texas MD Anderson Cancer Center (Supplementary Table S1). We also included samples from 20 healthy donors for comparison. We found that sEcad serum levels were significantly higher in IBC patients than in serum from healthy donors (*P *< .0001, Figure 1A). Having higher serum sEcad levels also correlated with metastasis status of IBC patients (*P *= .0036, Figure 1B), poor overall survival (OS; *P *= .0434, Figure 1C), reduced breast cancer-specific survival (*P *= .0434, Figure 1D), earlier onset of metastasis (*P *= .0045, Figure 1E), and an increased risk of developing brain metastasis (*P *= .0438, Figure 1F). However, higher serum sEcad levels were not correlated with the incidence of lung metastasis (*P *= .1135; Supplementary Figure S2A) but were correlated with the incidence of bone metastasis (*P *= .0122; Supplementary Figure S2B), suggesting that sEcad has a role in organ site-specific metastasis. Univariate analysis showed that sEcad levels, race, clinical disease stage, HR/HER2 status, lymphatic invasion, vascular invasion, and response to neoadjuvant chemotherapy were associated with overall survival and breast cancer-specific survival (Supplementary Table S2). On multivariable analysis, high sEcad levels independently predicted poor OS (hazard ratio [HR] =1.905, 95% confidence interval [CI]: 1.174-3.093, *P *= .009) and breast cancer-specific survival (HR = 1.898, 95% CI: 1.153-3.123, *P *= .0117), along with clinical disease stage and receptor status (Supplementary Table S3). These findings highlight the clinical significance of sEcad in patients with IBC and support its potential role in brain metastasis.

### sEcad Promotes IBC Cell Invasion, Migration, and Resistance to Anoikis

**Figure 2.**
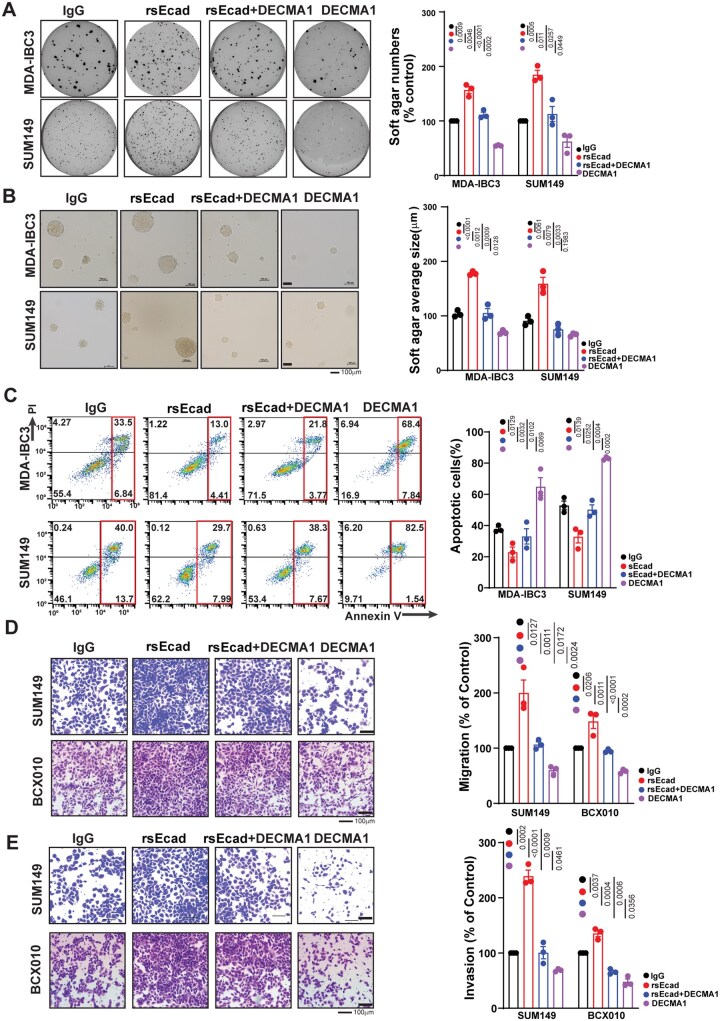
Soluble E-cadherin (sEcad) promotes inflammatory breast cancer (IBC)cell invasion, migration, and resistance to anoikis. IBC cells were treated with sEcad-recombinant protein (rsEcad) (20 µg/mL) in the presence or absence of DECMA1 neutralizing antibody (20 µg/mL). Data in (A-E) are mean ± SEM of 3 biological replicates. (A, B) rsEcad protein increased the (A) number and (B) size of anchorage-independent/soft agar-growing colonies of MDA-IBC3 and SUM149 cells, which was attenuated by treatment with DECMA1 (scale bar 100 µm). (C) rsEcad protein led to increased anoikis resistance in MDA-IBC3 and SUM149 cells, which was reversed by DECMA1. (D, E) rsEcad protein enhanced the (D) migration and (E) invasion of SUM149 and BCX010 IBC cells, which were counteracted by the neutralizing antibody DECMA1 (scale bar 100 µm).

During metastatic progression, tumor cells show increased migration and invasion and resistance to cell death. Therefore, we examined whether sEcad affects these traits in IBC cells. We treated cells with sEcad recombinant protein in the presence or absence of DECMA1, a monoclonal neutralizing antibody against the ectodomain of E-cadherin, which has been shown to inhibit sEcad.^43^ Treatment with sEcad recombinant protein (rsEcad) significantly increased the anchorage-independent growth of IBC cells [rsEcad vs IgG control (MDA-IBC3, *P *= .0009; SUM149, *P *= .0005)], which was counteracted by DECMA1 [rsEcad+ DECMA1 vs rsEcad (MDA-IBC3, *P *= .0046; SUM149, *P *= .011)] (Figure 2A). sEcad also increased the size of soft agar colonies relative to the control (MDA-IBC3, *P *< .0001; SUM149, *P *= .0061) (Figure 2B). These results indicate that sEcad-treated cells exhibit improved viability under non-adherent culture conditions. This anchorage-independent growth reflects the cancer cells’ ability to resist anoikis.^47^^,^^51^^,^^52^ Anoikis resistance, or the ability to escape apoptosis when detached from the extracellular matrix, is a crucial feature of cell survival during metastasis. Anoikis-resistant cells are known to form aggressive tumors that readily metastasize.^51^^,^^52^ Therefore, we examined the effects of sEcad on anoikis-mediated cell death in IBC cells. We found that treatment with recombinant sEcad significantly reduced cell death in IBC cells grown in poly-HEMA-treated low-attachment plates compared with untreated controls [rsEcad vs IgG control (MDA-IBC3, *P *= .0129; SUM149, *P *= .0139)], an effect that was reversed by DECMA1 [sEcad+DECMA1 vs sEcad (MDA-IBC3, *P *= .032; SUM149, *P *= .0252)] (Figure 2C). These results suggest that sEcad enhances the resistance of breast cancer cells to anoikis, potentially enabling them to survive in circulation. We then examined the effect of sEcad on the migration and invasion of IBC cells by using the cell lines SUM149 and BCX010, which we used previously for similar studies because IBC3 has minimal baseline or induced migration capacity.^14^ Cells treated with recombinant sEcad exhibited increased migration (rsEcad vs IgG control [SUM149, *P *= .0127; BCX010, *P *= .0206]) (Figure 2D) and invasiveness (rsEcad vs IgG control [SUM149, *P *= .0002; BCX010, *P *= .0037]) (Figure 2E). Collectively, these findings underscore the possible significance of sEcad in driving the metastatic progression of IBC tumors.

As noted above, published studies of sEcad in cancer have primarily used recombinant proteins; however, a more stable and convenient cell-based system is essential to investigate sEcad’s role in tumor growth and metastasis and elucidate its underlying mechanisms. To address this need, we cloned the 80-kDa sEcad fragment based on the full-length E-cadherin structure (Supplementary Figure S3A, left) into the Lenti-Blast vector and established stable Flag-tagged sEcad-overexpressing IBC cell lines [ER–/HER2+ (MDA-IBC3; SUM190) and ER–/HER2– (SUM149; BCX010)], which were validated by immunoblotting (Supplementary Figure S3A, right). Using these newly established sEcad-overexpressing stable cell lines, we examined sEcad’s role in invasion, migration, anchorage-independent growth, and anoikis resistance. The results mirrored those observed with the sEcad recombinant protein, with overexpression of sEcad leading to a significant increase in the number and size of soft agar colonies in all 4 IBC cell lines (Supplementary Figure S3B and C). Moreover, IBC cells overexpressing sEcad exhibited increased resistance to anoikis (Supplementary Figure S3D) as well as increased migration (Supplementary Figure S3E) and invasion (Supplementary Figure S3F) compared with controls. We further found that sEcad activated NF-κB signaling while suppressing cleaved caspase-3 (Supplementary Figure S3G), supporting its role in promoting resistance to cell death through the NF-κB pathway.

In addition to the effects of sEcad on invasion and anoikis resistance, we examined whether sEcad enhances tumor cell competence for other steps in the brain metastasis cascade, including endothelial cell adhesion, angiogenesis, and trans-endothelial migration. We found that sEcad-expressing IBC cells significantly increased tumor cell adhesion to endothelial cells (Supplementary Figure S4A) and promoted angiogenesis (Supplementary Figure S4B). Similar results were observed with sEcad recombinant protein, and these effects were neutralized by DECMA1 (Supplementary Figure S4C). Moreover, in our in vitro blood-brain barrier (BBB) trans-endothelial migration model, sEcad-overexpressing SUM149 cells demonstrated greater penetration across the BBB than the control (Supplementary Figure S4D), suggesting that sEcad promotes the ability of tumor cells to invade the central nervous system tissue. Collectively, these findings validate our newly generated stable sEcad-overexpressing cell lines as a reliable model for studying the role of sEcad in tumor growth and metastasis. The results replicate our findings from the recombinant protein studies while providing a more stable, reproducible, and efficient system for mechanistic studies and animal experiments.

### sEcad Is a Driver of Brain Metastasis in IBC Models

**Figure 3.**
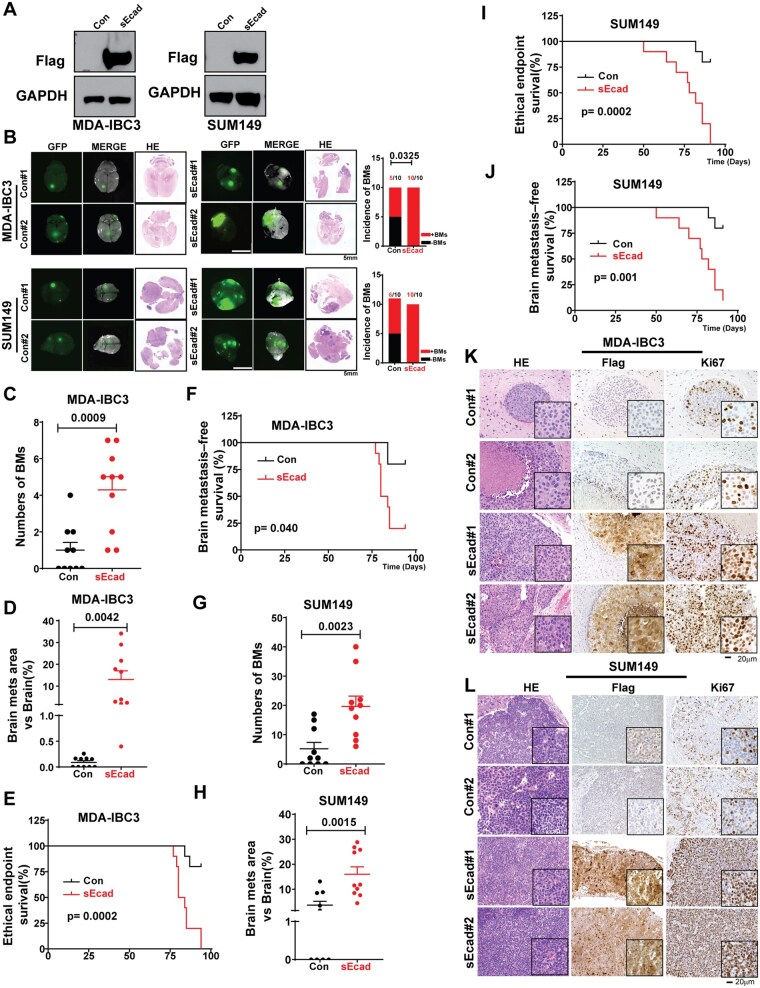
Soluble E-cadherin (sEcad) is a driver of brain metastasis in inflammatory breast cancer (IBC) models. (A) Generation of sEcad-FLAG-overexpressing MDA-IBC3 and SUM149 stable IBC cell lines. Total cell lysates were analyzed by western blotting with anti-FLAG and anti-GAPDH antibodies. (B-L) For brain metastasis studies, HER2+ MDA-IBC3 cells (1 × 10^6^) were injected via tail vein, and the triple-negative IBC cell line SUM149 (2 × 10^5^) were injected via intracardiac injection into SCID/Beige mice. (B) (left) Representative images of brain metastases from sEcad-expressing and control MDA-IBC3 and SUM149 cells (scale bar 5 mm); (right) incidence of brain metastases in sEcad-overexpressing MDA-IBC3 and SUM149 cells compared with controls. (C, G) Numbers of brain metastases in sEcad-overexpressing group compared with controls. (D, H) Size of brain metastasis lesions in sEcad-overexpressing group compared with controls. (E, I) Overall survival rates in mice injected with sEcad-overexpressing or control IBC cells. (F, J) Brain metastasis-free survival rates in mice injected with sEcad-overexpressing or control IBC cells. (K, L) Hematoxylin-and-eosin and immunohistochemical staining of brain metastasis lesions from MDA-IBC3 and SUM149 control cells or sEcad-overexpressing cells. Flag indicates sEcad overexpression, whereas Ki67 indicates proliferation in brain metastasis lesions from the 2 mouse models of IBC brain metastasis (scale bar 20 µm).

To determine the role of sEcad in brain metastatic progression, we used 2 IBC cell line models with a high propensity for brain metastasis (MDA-IBC3 and SUM149)^53^ with and without sEcad-Flag stable expression (Figure 3A). Mice injected via tail-vein with sEcad-overexpressing MDA-IBC3 cells had a higher incidence of brain metastasis compared with controls (MDA-IBC3-sEcad vs MDA-IBC3-Con: 100% vs 50%, *P *= .032; Figure 3B). sEcad-overexpressing IBC cells also formed larger, grossly visible brain metastases and increased metastasis number (MDA-IBC3: *P *= .0009, Figure 3C) and size (MDA-IBC3: *P *= .0042, Figure 3D). Further, sEcad reduced the overall survival of mice (Figure 3E, *P *= .0002) and shortened brain metastasis-specific survival time (MDA-IBC3: *P *= .04, Figure 3F). In a second model, mice injected intracardially with sEcad-overexpressing SUM149 cells also had a higher incidence of brain metastasis (100% vs 60%; Figure 3B, lower panel), increased number of metastases (*P *= .0023, Figure 3G), increased size of metastases (*P *= .0015, Figure 3H), and reduced overall survival (*P *= .0002, Figure 3I) and brain metastasis-free survival (*P *= .001, Figure 3J) compared with controls. Neither of these IBC models metastasized to the bone or other organs. Representative hematoxylin-and-eosin and immunohistochemically stained images of Flag and Ki67 (Figure 3K and L) confirmed sEcad overexpression and increased proliferation in the brain metastasis lesions in both the MDA-IBC3 and SUM149 models. These findings support that sEcad is a driver of brain metastasis in IBC mouse models.

### sEcad Triggers Astrocyte Reactivity

The anoikis resistance observed in rsEcad-treated and sEcad-overexpressing IBC cells (Figure 2, Supplementary Figure S3) could be associated with increased overall metastasis. However, our patient and preclinical in vivo data (Figures 1 and 3) suggest that sEcad preferentially drives brain metastases. This led us to hypothesize that in addition to cell-autonomous induction of anoikis resistance, sEcad may have paracrine effects on the brain microenvironment, inducing a supportive niche for metastatic breast cancer growth. Increasing evidence suggests that reactive astrocytes, marked by high levels of glial fibrillary acidic protein (GFAP), foster brain metastasis growth and progression via modulation of the BBB, promotion of an inflammatory and immunosuppressive brain microenvironment, enrichment of cancer stem cells, and increasing proliferation and invasion of metastatic cancer cells.^54–59^ To determine whether sEcad induces reactive astrocytosis, we treated an immortalized normal human astrocyte (NHA) cell line with recombinant sEcad (20 µg/mL for 36 hours) or an IgG control. Immunofluorescence and immunoblotting analysis showed that sEcad significantly increased GFAP protein expression (Supplementary Figure S5A and B), indicating direct induction of reactive astrocytes by sEcad in vitro. In vivo, tail-vein injection of sEcad-overexpressing MDA-IBC3 cells led to a similar significant increase in GFAP-positive astrocytes within brain metastatic lesions relative to controls (Supplementary Figure S5C). Similarly, intracardiac injection of SUM149-sEcad cells resulted in increased reactive astrocyte induction in brain tissue lesions compared with controls (Supplementary Figure S5D). These findings were further validated by immunohistochemical staining with an anti-GFAP antibody (Supplementary Figure S5E and F). These results suggest that tumor-secreted sEcad acts on the microenvironment to promote a metastatic niche in the brain via induction of reactive astrocytes.

### sEcad-High Cells Induce CXCR2 Activation via CXCL1/CXCL8

**Figure 4.**
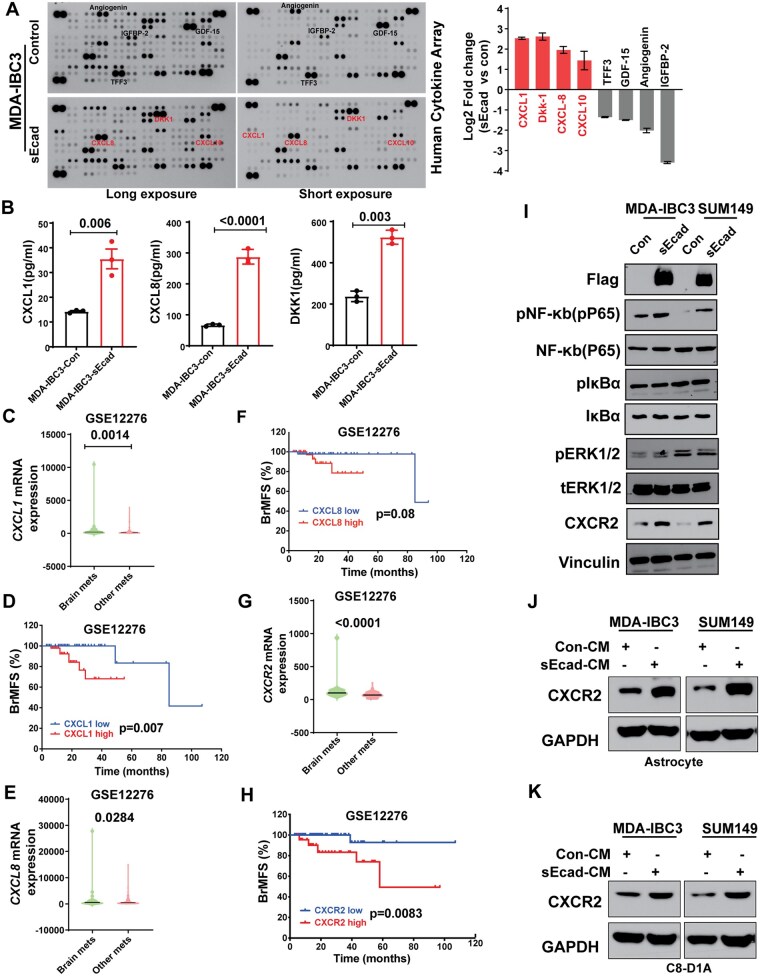
Soluble E-cadherin (sEcad)-overexpressing cells induce astrocyte and CXCR2 activation via CXCL1/CXCL8. (A, B) Cytokine array analysis of secreted factors showed increased levels of the pro-inflammatory cytokines CXCL1/CXCL8 in conditioned medium from sEcad-overexpressing MDA-IBC3 cells, as validated by ELISA. (C) CXCL1 was expressed at higher levels in patients with brain metastasis than in patients with metastasis to other anatomic sites. (D) Patients with high CXCL1 expression had reduced brain metastasis-free survival (BrMFs, GSE12276); CXCL1-low indicates the bottom (25th) tertile, and CXCL1-high the top (75th) tertile. (E) CXCL8 was expressed at higher levels in patients with brain metastasis relative to patients with metastasis to other anatomic sites. (F) Patients with high CXCL8 expression had reduced BrMFs (GSE12276); CXCL8-low indicates the bottom (25th) tertile, and CXCL8-high the top (75th) tertile. (G) CXCR2 was expressed at higher levels in patients with brain metastasis than in patients with metastasis to other anatomic sites. (H) Patients with high CXCR2 expression had reduced BrMFs (GSE12276); CXCR2-low indicates the bottom (25th) tertile, and CXCR2-high the top (75th) tertile. (I) Western blot showing increased NF-κB p65 phosphorylation and CXCR2 expression in sEcad-overexpressing MDA-IBC3 and SUM149 cells; *v*inculin is shown as a loading control. (J, K) Induction of CXCR2 expression in human astrocytes (J) and mouse astrocytes (K) treated with 30% conditioned medium from sEcad-overexpressing cells. GAPDH served as a loading control.

To elucidate the mechanisms by which sEcad can lead to modification of local microenvironments (in the brain or elsewhere), we used cytokine array analysis of secreted factors in conditioned medium collected from sEcad-expressing and control SUM149 and MDA-IBC3 cells. We found a significant increase in pro-inflammatory cytokines (CXCL1, CXCL8, and CXCL10) and secretory proteins (DKK1) in conditioned medium from sEcad-expressing cells (Figure 4A, Supplementary Figure S6A). These findings were validated by ELISA (Figure 4B, Supplementary Figure S6B).

**Figure 5.**
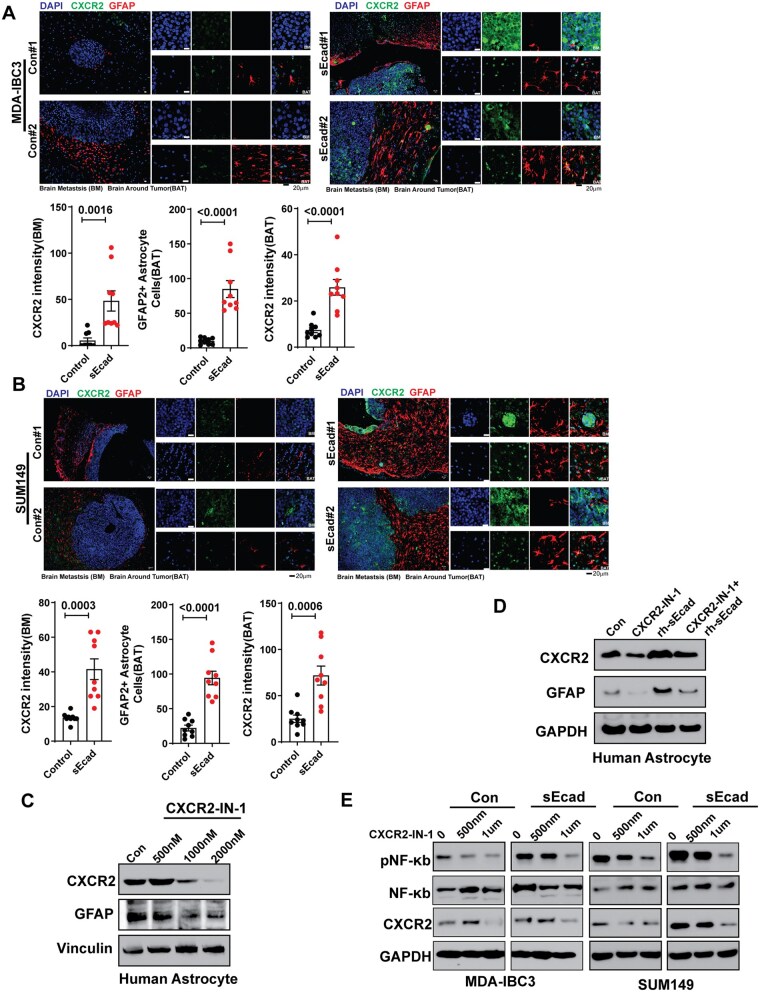
Soluble E-cadherin (sEcad) induces reactive astrocytes and CXCR2. (A, B) Immunofluorescence staining showed that metastatic lesions from sEcad-expressing tumors had significantly higher GFAP+ reactive astrocytes and CXCR2 expression than did the Control groups. Quantification of CXCR2, GFAP-positive astrocytes. Data are mean ± SEM, *n* = 3 mice and 3 fields per mouse: *t* test (scale bar 20 µm). (C) Western blotting shows that the CXCR2 inhibitor CXCR2-IN-1 decreased CXCR2 and GFAP levels in human astrocytes; Vinculin served as a loading control. (D) Inhibition of CXCR2 and GFAP by CXCR2-IN-1 could be rescued by sEcad-recombinant protein (rsEcad). GAPDH served as a loading control. (E) sEcad increased the expression of CXCR2 and phosphorylated NFκB (pP65) in IBC cells, both of which were inhibited by CXCR2-IN-1. GAPDH served as a loading control.

To prioritize these based on clinical relevance, we analyzed gene expression data obtained from patients with metastatic breast cancer (GEO dataset GSE12276).^60^ We found that the expression of CXCL1 and CXCL8, but not DKK1, was significantly higher in primary tumors from patients who developed brain metastases compared with primary tumors that developed other metastases (Figure 4C and E, Supplementary Figure S7A). Moreover, having high CXCL1 or CXCL8 expression, but not DKK1 expression, was correlated with reduced brain metastasis-free survival (Figure 4D and F, Supplementary Figure S7B), suggesting that CXCL1 and CXCL8 may be related to sEcad’s promotion of brain metastases. Notably, expression of the CXCR2 receptor, a common receptor for both CXCL1 and CXCL8, was also significantly higher in patients with brain metastases and correlated with reduced brain metastasis-free survival (Figure 4G and H). These findings were intriguing given that CXCR2 is expressed in cancer cells, astrocytes, and microglia,^61^ suggesting that the CXCL1/CXCL8-CXCR2 axis may have a pivotal role in the brain-metastatic preference and progression of sEcad-expressing IBC cells. Consistent with these findings, we found increased expression of CXCR2 in cancer cells (Figure 4I) and in human and mouse astrocytes (Figure 4J and K) treated with conditioned medium from sEcad-overexpressing IBC cells compared with those treated with conditioned medium from control cells. CXCR2 and GFAP levels were consistently elevated in brain metastases and surrounding brain parenchyma from mice injected with sEcad-high cells compared with controls (Figure 5A and B). We further investigated how sEcad may activate CXCL1-CXCR2 signaling in IBC cells and astrocytes. We found that sEcad increased phospho-p65 but not p-IκBα or pERK1/2 (Figure 4I), suggesting that sEcad enhances NF-κB activation via phosphorylation of the p65 subunit. Recombinant sEcad similarly activated NF-κB in tumor cells and astrocytes (Supplementary Figure S8A). Consistent with this, treatment of sEcad-expressing MDA-IBC3 and SUM149 cells with the p65 inhibitor JSH-23 significantly reduced CXCL1, CXCL8, phospho-p65, and CXCR2 (Supplementary Figure S8B-D), indicating that NF-κB/p65 activation is required for these effects in tumor cells. To demonstrate that sEcad activates CXCR2, we measured cAMP levels as a functional readout of Gαi-coupled G-protein-coupled receptor (GPCR) signaling.^62^ Recombinant sEcad reduced cAMP levels in MDA-IBC3 and SUM149 cells as well as in astrocytes (Supplementary Figure S9A), consistent with the established role of Gi-linked CXCR2 in suppressing adenylyl cyclase activity.^63^ In sEcad-overexpressing MDA-IBC3 and SUM149 cells, treatment with the CXCR2 antagonists SB225002 and CXCR2-IN-1 reversed this reduction (Supplementary Figure S9B), confirming that the observed effects depend on CXCR2. In addition, sEcad increased p-CXCR2, total CXCR2, β-arrestin1/2, and p-p65 (Supplementary Figure S8A). CXCR2 phosphorylation and β-arrestin recruitment are well-established markers of receptor activation^64–66^ further supporting the conclusion that sEcad engages CXCR2 signaling. Taken together, these results demonstrate that sEcad drives an NF-κB–CXCL1/CXCL8-CXCR2 tumor–astrocyte signaling loop that promotes brain metastasis.

### CXCR2 Inhibitor Significantly Reduces Brain Metastasis and Prolongs Survival

To examine if sEcad promotes reactive astrocytes and brain metastases via a CXCL1/CXCL8-CXCR2-related mechanism, we inhibited this axis with CXCR2-IN-1, a brain-penetrant inhibitor of CXCR2,^67^ that may have potential as a pharmacologic tool for studying brain metastasis. Treatment of NHAs with various doses of CXCR2-IN-1 resulted in reduced expression of CXCR2 and GFAP, as confirmed by western blotting and immunofluorescence (Figure 5C, Supplementary Figure S10A). Further, CXCR2-IN-1 reduced GFAP+ reactive astrocytes induced by sEcad recombinant protein (Figure 5D, Supplementary Figure S10B). Similar results were obtained with another CXCR2 antagonist, SB225002, which effectively inhibited CXCR2 and GFAP expression (Supplementary Figure S10C). In sEcad-high IBC cells, both CXCR2-IN-1 and SB225002 suppressed the phosphorylation of NF-κB (p65) (Figure 5E, Supplementary Figure S10D) and CXCR2 activation (Supplementary Figure S9B). We also assessed the effect of CXCR2-IN-1 on tumor-cell behavior in vitro. In sEcad-overexpressing MDA-IBC3 and SUM149 cells, CXCR2-IN-1 treatment significantly reduced colony formation, migration, and invasion (Supplementary Figure S11A-D). We observed similar inhibitory effects in the corresponding control cells, underscoring a consistent role for CXCR2 signaling in driving pro-oncogenic and pro-metastatic behavior of IBC cells. Collectively, these data identify CXCR2-IN-1 as an effective pharmacologic inhibitor of reactive astrocytosis and tumor cell proliferation, migration, and invasion.

**Figure 6.**
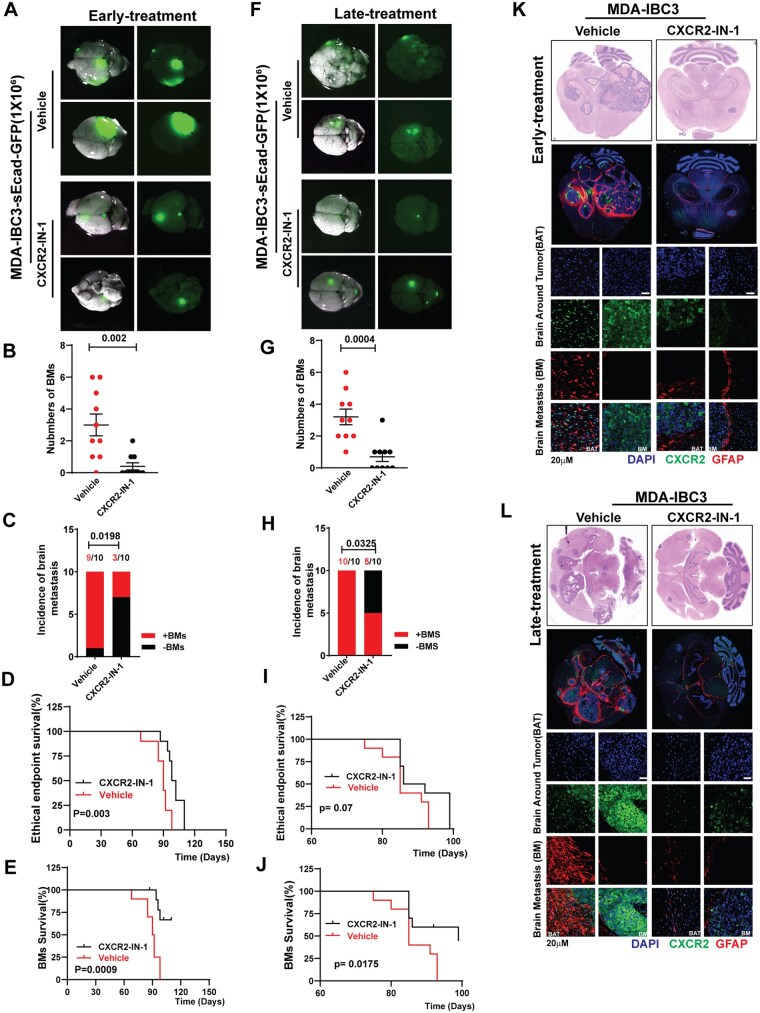
CXCR2 inhibitor reduces brain metastasis and prolongs survival. The schema for the “early” and “late treatment” approaches for the MDA-IBC3 brain metastasis model is shown in Supplementary Figure S12A. (A-E) In the “early treatment” group, CXCR2-IN-1 reduced brain metastases in MDA-IBC3-sEcad-injected mice. (A) Representative images show reduced brain metastasis in CXCR2-IN-1-treated MDA-IBC3-sEcad mice (scale bar 5 mm). CXCR2-IN-1 reduced the number of brain metastasis lesions (B) and incidence of metastasis (C) and improved overall survival (D) and brain metastasis-free survival (E). (F-J) In the “late treatment” group, (F) representative images show reduced brain metastasis in CXCR2-IN-1-treated brain metastasis-bearing MDA-IBC3-sEcad (scale bar 5 mm). CXCR2-IN-1 reduced the number of brain metastasis lesions (G), incidence of metastasis (H) and improved overall survival (I) and brain metastasis-free survival (J) in mice. (K, L) Hematoxylin-and-eosin and immunofluorescence analyses of brain metastases show reduced reactive astrocytes (GFAP+) and CXCR2 expression in both “early group” (K) and “late” (L) CXCR2-IN-1 treatment groups (scale bar 20 µm).

To investigate if CXCR2-IN-1 can similarly inhibit the growth and progression of sEcad-expressing IBC in our brain metastasis models, we implemented 2 treatment strategies: an “early treatment” approach targeting microscopic metastases and a “late treatment” approach targeting macroscopic metastases (Supplementary Figure S12A and B). In the “early treatment” group, CXCR2-IN-1 significantly reduced brain metastases in both MDA-IBC3-sEcad and SUM149-sEcad models (Figure 6A, Supplementary Figure S13A). Quantitative analysis revealed significant decreases in the number of brain metastases per mouse (Figure 6B, Supplementary Figure S13B) and the incidence of brain metastasis (Figure 6C, Supplementary Figure S13C). CXCR2-IN-1 treatment also led to improved overall survival (Figure 6D, Supplementary Figure S13D) and brain metastasis-free survival (Figure 6E, Supplementary Figure S13E) in both models. In the “late treatment” group, CXCR2-IN-1 significantly reduced brain metastases in both models (Figure 6F, Supplementary Figure S13F). Analysis of vehicle- and CXCR2-IN-1-treated mice showed reductions in brain metastasis number (Figure 6G, Supplementary Figure S13G) and incidence (Figure 6H, Supplementary Figure S13H), in addition to prolonged overall survival (Figure 6I, Supplementary Figure S13I), and brain metastasis-free survival (Figure 6J, Supplementary Figure S13J).

We evaluated the effect of CXCR2-IN-1 on CXCR2 expression and GFAP+ reactive astrocytes in vivo by using hematoxylin and eosin and immunofluorescence staining of brain metastases from vehicle-treated and CXCR2-IN-1-treated groups. In the “early treatment” group, CXCR2-IN-1 significantly reduced CXCR2 expression within brain metastases and the surrounding brain parenchyma in both MDA-IBC3-sEcad and SUM149-sEcad models (Supplementary Figure S14A and B). The proportions of reactive astrocytes were markedly decreased as well (Figure 6K, Supplementary Figures S13K, S14A and B). Similar results were observed in the “late treatment” group, where CXCR2 expression and GFAP+ reactive astrocytes were reduced in CXCR2-IN-1-treated lesions compared with vehicle-treated controls ­(Figure 6L, Supplementary Figures S13L, S14C and D). Representative hematoxylin and eosin stains showing results of our treatment strategy are shown in Supplementary Figure S14E and F. Together, these findings demonstrate that CXCR2-IN-1 significantly reduces brain metastases and prolongs survival even in established, gross disease, further supporting its potential as an effective therapeutic agent for preventing brain metastases in high-risk patients and treating established brain metastases. These findings suggest that CXCR2-IN-1 modulates the tumor microenvironment by suppressing the sEcad-mediated activation of reactive astrocytes (Supplementary Figure S15).

## Discussion

The molecular mechanisms driving breast cancer cell metastasis to the brain and the interaction of metastases with the brain microenvironment remain poorly understood, which hinders progress in developing new treatments. In this study, we identified sEcad as an indicator of poor prognosis associated with increased risk of brain metastasis in patients with IBC. By using novel xenograft IBC mouse models, we demonstrated that sEcad is a driver of brain metastasis. sEcad from IBC tumor cells promotes cancer cell invasion and anoikis resistance and activates NF-κB signaling. Tumor-secreted sEcad further upregulates CXCR2 in astrocytes, which respond to sEcad-expressing tumor-cell secretion of CXCR2 ligands to promote the metastatic brain niche. Inhibition of CXCR2 blocks sEcad-induced pro-metastatic phenotypes, and notably, treating sEcad-high brain metastases with a brain-permeable CXCR2 inhibitor reduced metastasis burden and prolonged survival in IBC mouse models. Collectively, our findings highlight the dual role of sEcad in the brain metastatic cascade: (i) promoting invasion and anoikis resistance in breast cancer cells, potentially via NF-κB signaling, and (ii) fostering a supportive brain microenvironment through astrocyte activation and CXCL1/CXCL8-CXCR2 signaling.

One defining feature of metastasis is the dynamic regulation of cadherins, key adhesion molecules that drive cell growth, invasion, and migration. Although E-cadherin is often associated with low metastatic potential,^10^^,^^11^ aggressive tumors like IBC paradoxically overexpress it, and its depletion reduces invasion and tumor growth in in vitro and in vivo models of IBC.^14^^,^^20–22^ However, sEcad, the cleaved 80-kDa fragment of E-cadherin, has been linked to tumor progression in several types of cancer and is highly expressed in the serum of cancer patients, correlating with poor prognosis.^24^^,^^28^^,^^37–46^ In breast cancer, high serum sEcad levels are associated with increased metastatic risk and worse outcomes,^68–71^ emphasizing the need to understand its role in metastasis. Prior studies relied on recombinant sEcad protein or neutralizing antibodies, thereby limiting long-term survival and metastasis studies. To address this, we cloned sEcad and generated stable sEcad-expressing cell lines to provide a more robust tool for investigating its function in IBC survival, tumor growth, and brain metastasis. Findings from these sEcad-high stable cell lines mirrored those from recombinant sEcad protein, both demonstrating sEcad’s pro-tumorigenic and pro-metastasis roles in IBC models.

Notably, although our observations strongly imply that sEcad/E-cadherin directly promotes metastasis in IBC, our findings may have broader relevance to other tumor types characterized by elevated levels of these molecules. For example, in prostate cancer, elevated serum sEcad has been associated with advanced metastatic stage, and E-cadherin expression correlates positively with invasive growth and bone metastasis.^24–26^ In ovarian cancer, over 80% of tumors display high E-cadherin expression, which is associated with poor outcomes and enhanced angiogenesis and invasion of ovarian epithelial cells.^27^^,^^28^ In gastric cancer as well, elevated levels of sEcad are associated with increased invasion and reduced survival.^29–31^ Similarly, E-cadherin has been linked to poor prognosis in patients with glioblastoma.^32^ Collectively, these findings suggest that the oncogenic functions of sEcad/E-cadherin extend beyond IBC to a range of epithelial cancers and taken together with the findings presented here, imply a new mechanism by which E-cadherin promotes aggressiveness across cancers.

Examining mechanisms underlying sEcad’s propensity for brain metastasis, we found that sEcad induces reactive astrocytosis in the brain microenvironment by increasing the release of pro-inflammatory cytokines from tumor cells, including CXCL1 and CXCL8, and activating their receptor, CXCR2, in astrocytes. In this previously unreported mechanism, sEcad-high cancer cells exploit the CXCL1/CXCL8-CXCR2 axis to create a supportive niche for brain metastasis growth. We further demonstrated that sEcad activates NF-κB/p65 signaling, leading to increased CXCL1/CXCL8 expression and CXCR2 upregulation in both cancer cells and astrocytes. CXCR2 is involved in various neurological conditions, including neurological repair in multiple sclerosis, optic nerve injury, and neuropathic pain; it is also expressed in immune cells, endothelial cells, and mesenchymal stem cells.^72–78^ In brain injury and autoimmune disease models, CXCR2 participates in maintaining BBB integrity,^79^^,^^80^ whereas in brain metastasis it has been shown to reprogram neutrophils to promote tumor progression.^81^ Given the growing evidence that reactive astrocytes foster brain metastasis by compromising the BBB,^54–59^ thus allowing easier access for tumor cells, we investigated whether sEcad-mediated brain metastasis and astrocyte activation are driven by CXCR2. To the best of our knowledge, no prior studies have explored CXCR2-mediated astrocyte activation as a mechanism for brain metastasis, and our findings strongly support this hypothesis through both in vitro studies and IBC brain metastasis models. We further demonstrate that the CXCR2 antagonist CXCR2-IN-1 effectively blocks brain metastasis and prolongs survival in preclinical mouse models. Published studies^81^^,^^82^ have shown that CXCR2 signaling promotes metastatic growth in breast cancer, thereby reinforcing our observations. These findings highlight inhibition of CXCR2 as a potential therapeutic strategy that should be investigated more extensively in preclinical and clinical studies of metastatic brain progression.

In summary, this study reveals sEcad as a key driver of brain metastasis in IBC, demonstrating its critical role in several steps in the metastatic cascade. We demonstrate that sEcad not only enhances tumor cell-intrinsic survival but also reshapes the brain microenvironment by activating astrocytes, fostering a pro-metastatic niche that drives selective brain colonization. Our findings that we can achieve inhibition of brain metastasis with a BBB-penetrant inhibitor of CXCR2 highlight the therapeutic potential of targeting the sEcad-CXCL1-CXCR2 pathway in aggressive cancers. Disrupting this axis is a promising strategy to limit brain metastasis in IBC and other malignancies that metastasize to the brain.

## Supplementary Material

noag012_Supplementary_Data

## Data Availability

Further information and requests for resources and reagents should be directed to and will be fulfilled by the lead contact, B.G.D. (bgdebeb@mdanderson.org). Plasmids generated in this study are available upon reasonable request from the corresponding author.
