# Supplementary material for: Soluble E-cadherin-CXCL1-CXCR2 axis as a therapeutic vulnerability in inflammatory breast cancer brain metastasis
Source: Neuro Oncol. 2026 Jan 23;28(5):1220–36. doi: 10.1093/neuonc/noag012 (PMC13158929; doi:10.1093/neuonc/noag012)
Supplement: noag012_Supplementary_Data [file noag012_supplementary_data.zip › SI Figures and Tables.docx]

**Soluble E-cadherin–CXCL1–CXCR2 Axis as a Therapeutic Vulnerability in Inflammatory Breast Cancer Brain Metastasis**

**Xiaoding Hu,****^1,7^ Yun Xiong,^2^ Emilly S Villodre,^1,7^ Huimin Zhang,^2^ Isabella R Longa^1,7^, Juhee Song,^3^ Natalie Fowlkes,^4^ Savitri Krishnamurthy,^5,7^ Marissa Rylander,^8^ Chandra Bartholomeusz,**^1,7^ **Debu Tripathy,^1 7^ Wendy A Woodward,^6,7^ Junjie Chen,^2^ and Bisrat G Debeb^1,7,*^**

Departments of ^1^Breast Medical Oncology, ^2^Experimental Radiation Oncology, ^3^Biostatistics ,^4^ Veterinary Medicine and Surgery, ^5^Pathology, ^6^Breast Radiation Oncology, and ^7^MD Anderson Morgan Welch Inflammatory Breast Cancer Clinic and Research Program, The University of Texas MD Anderson Cancer Center, Houston TX.^8^Department of Biomedical Engineering, The University of Texas at Austin, Austin, TX

**SUPPLEMENTARY INFORMATION**

**Supplementary Figures S1-S15**

**Supplementary Tables S1-3**

**Supplementary Figures S1-S15**


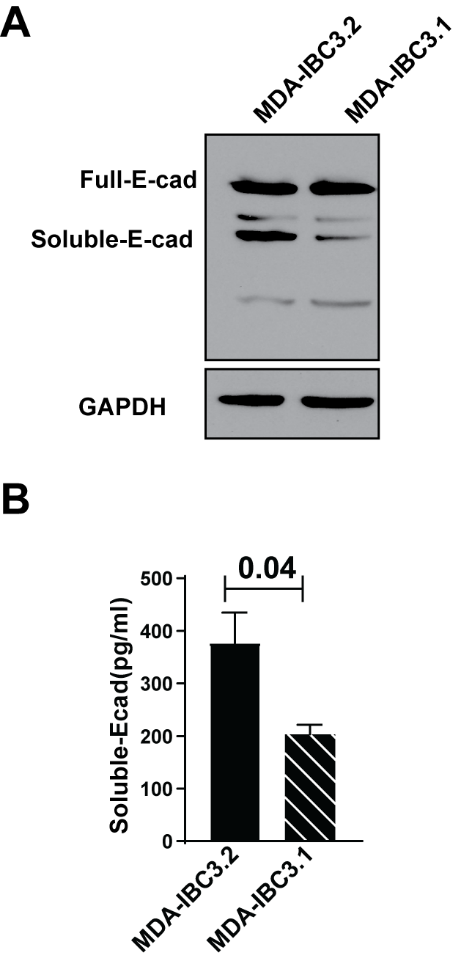


**Supplementary Figure S1. sEcad expression levels are higher in brain-metastasizing cell lines.**

(A) Western blot and (B) enzyme-linked immunosorbent assay findings show higher expression of sEcad in the highly brain metastasizing MDA-IBC3.2 cells compared with the weakly brain metastasizing MDA-IBC3.1 cells.

**
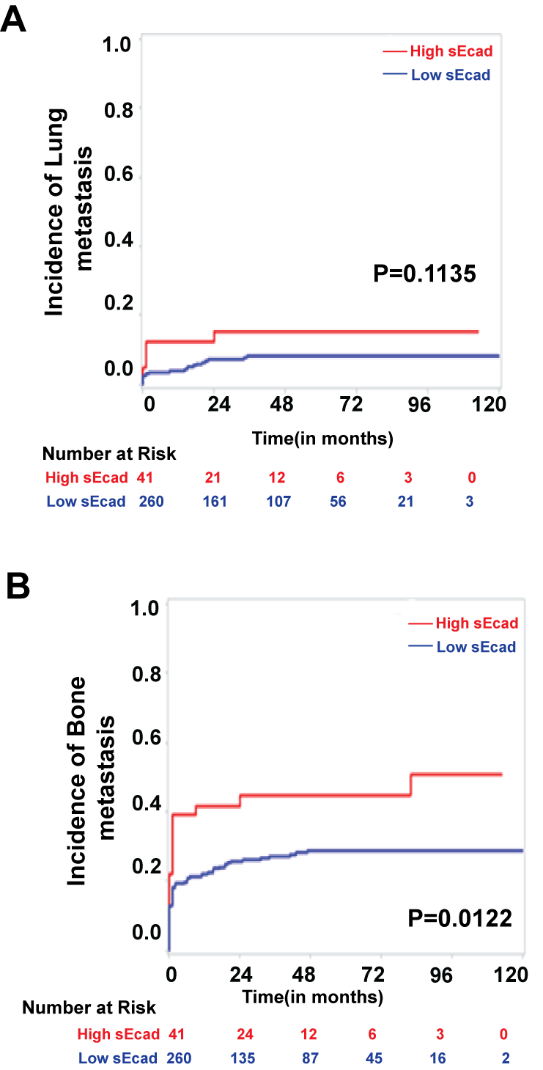
**

**Supplementary Figure S2. Serum sEcad levels and correlation with incidence of bone and lung metastasis.** (A) Kaplan–Meier analysis shows no significant difference in lung metastasis incidence between high- and low-sEcad groups (*P*=0.1135). (B) Bone metastasis incidence is higher in patients with higher levels of sEcad than in group with low-sEcad (*P*=0.0122).


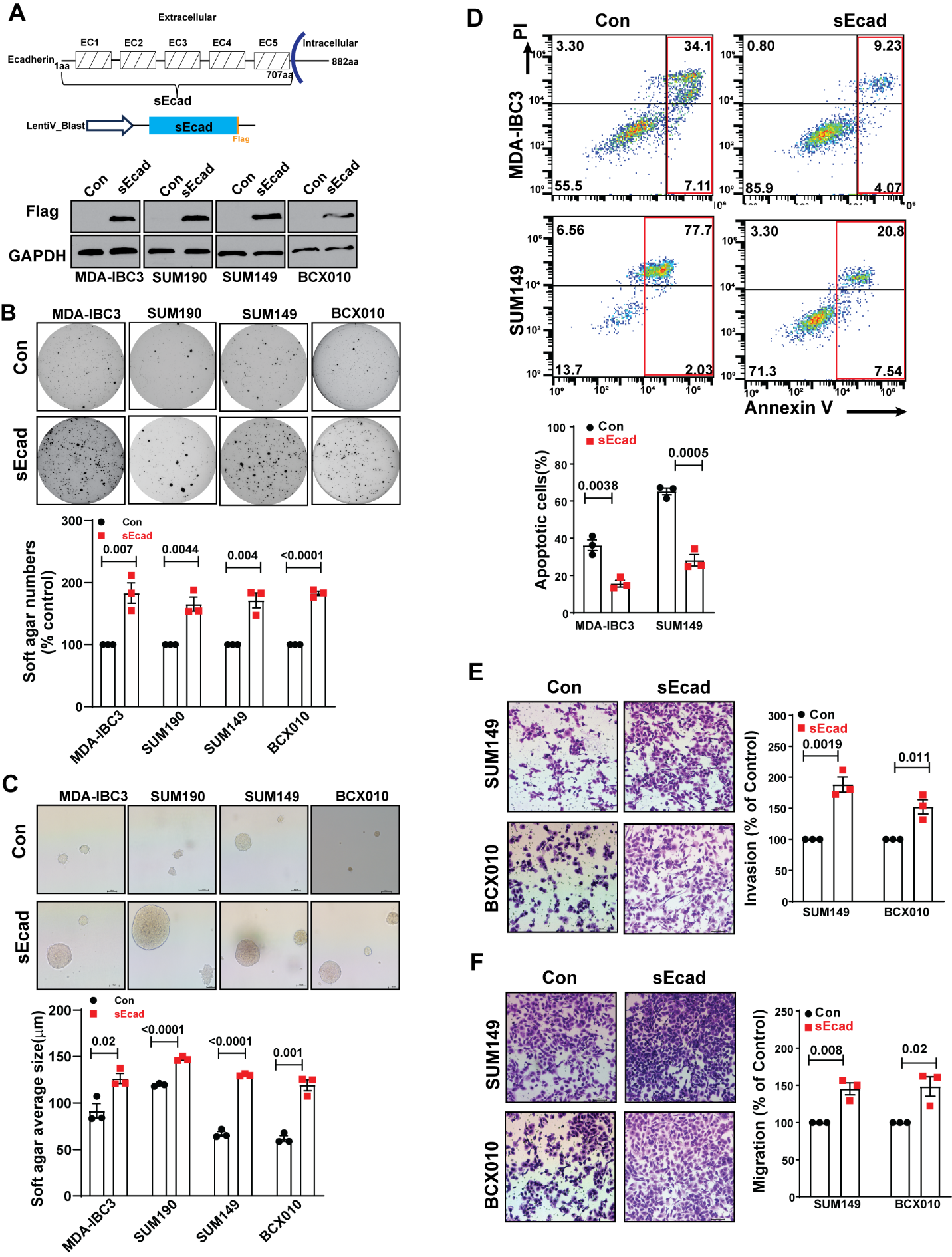


**Supplementary Figure S3. Ectopic overexpression of sEcad promotes anchorage-independent growth, migration, invasion, and anoikis resistance in IBC cells in vitro.** (A) Generation of sEcad-FLAG-overexpressing IBC stable cell lines. The total cell lysates of the 4 IBC cell lines (MDA-IBC3, SUM190, SUM149, BCX010) were analyzed by western blotting with anti-FLAG and anti-GAPDH (internal control) antibody. (B, C) sEcad overexpression increased the number (B) and size (C) of soft agar colonies in IBC cells. (D) sEcad overexpression inhibits anoikis in IBC cells. Control or sEcad over-expressing MDA-IBC3 and SUM149 cells were treated with poly-HEMA; 24 h later, cells were harvested and analyzed by flow cytometry with an FITC-Annexin V/PI- kit. (Right) Representative fluorescence-activating cell sorting analysis with (left) quantification of three independent experiments. (E) sEcad promotes migration of SUM149 and BCX010 IBC cells. (F) sEcad promotes invasion of SUM149 and BCX010 IBC cells. (G) Immunoblotting shows that sEcad activated NF-kB signaling and inhibited the cleavage of caspase-3 in IBC cells.


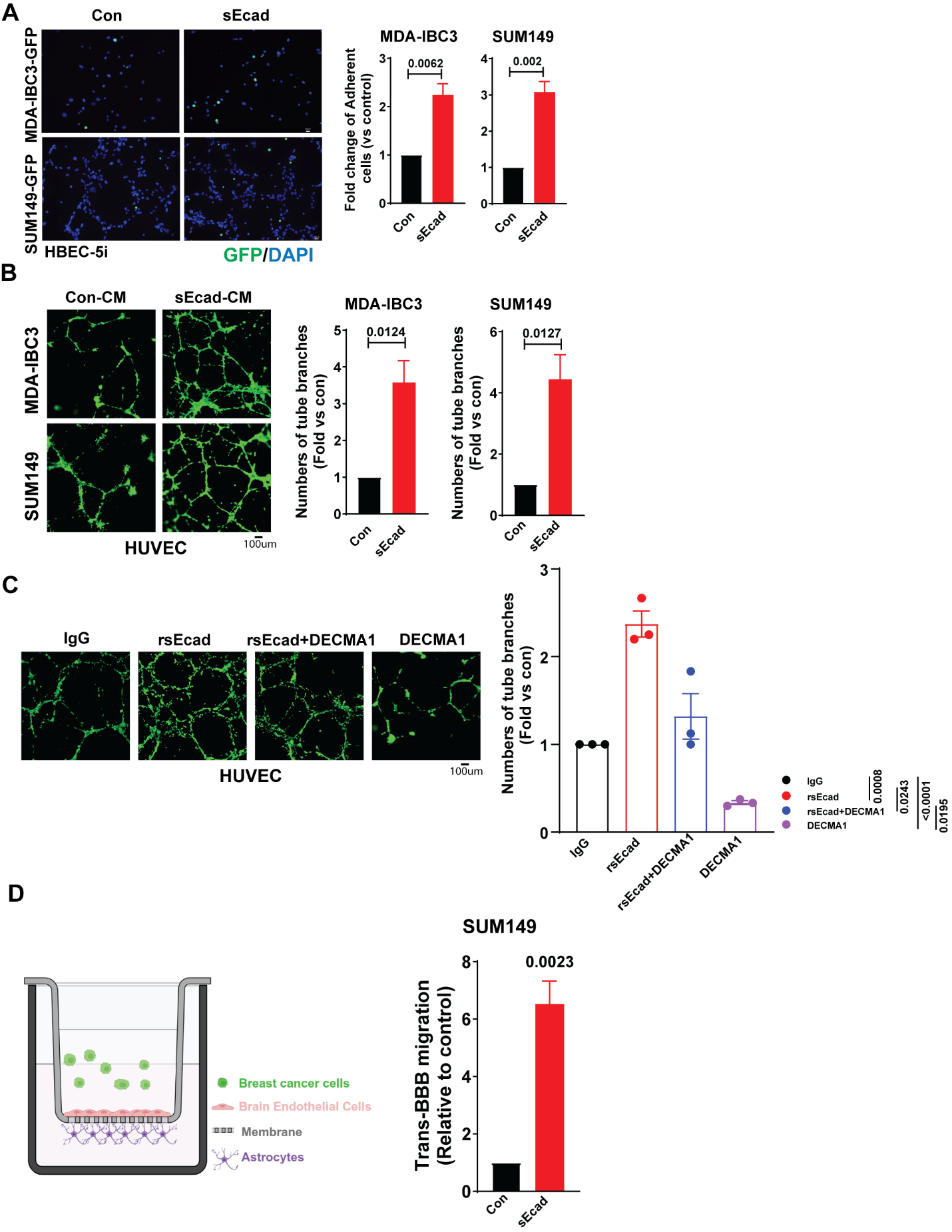


**Supplementary Figure S4. sEcad promotes in vitro endothelial cell adhesion, angiogenesis, and trans-endothelial migration.** (A) sEcad promotes brain endothelial cell adhesion. GFP-labeled sEcad-overexpressing IBC cells showed enhanced adhesion to human brain microvascular endothelial cells (HBEC-5i) in vitro compared with control cells. Data are represented as mean ± SEM from at least three independent experiments. (B) In vitro angiogenesis assay of human umbilical vein endothelial cells (HUVECs) treated with conditioned medium from MDA-IBC3 or SUM149 control and sEcad-overexpressing cells. Data on the right represent mean ± SEM of three biological replicates. (C) sEcad protein promotes angiogenesis, an effect countered by DECMA1. Quantification data represent mean ± SEM of three biological replicates. (D) In vitro BBB trans-endothelial migration model showed that (left) sEcad-overexpressing SUM149 cells had a higher migrating ability across the trans-BBB assay, with (right) quantification of trans-BBB migrating cells.


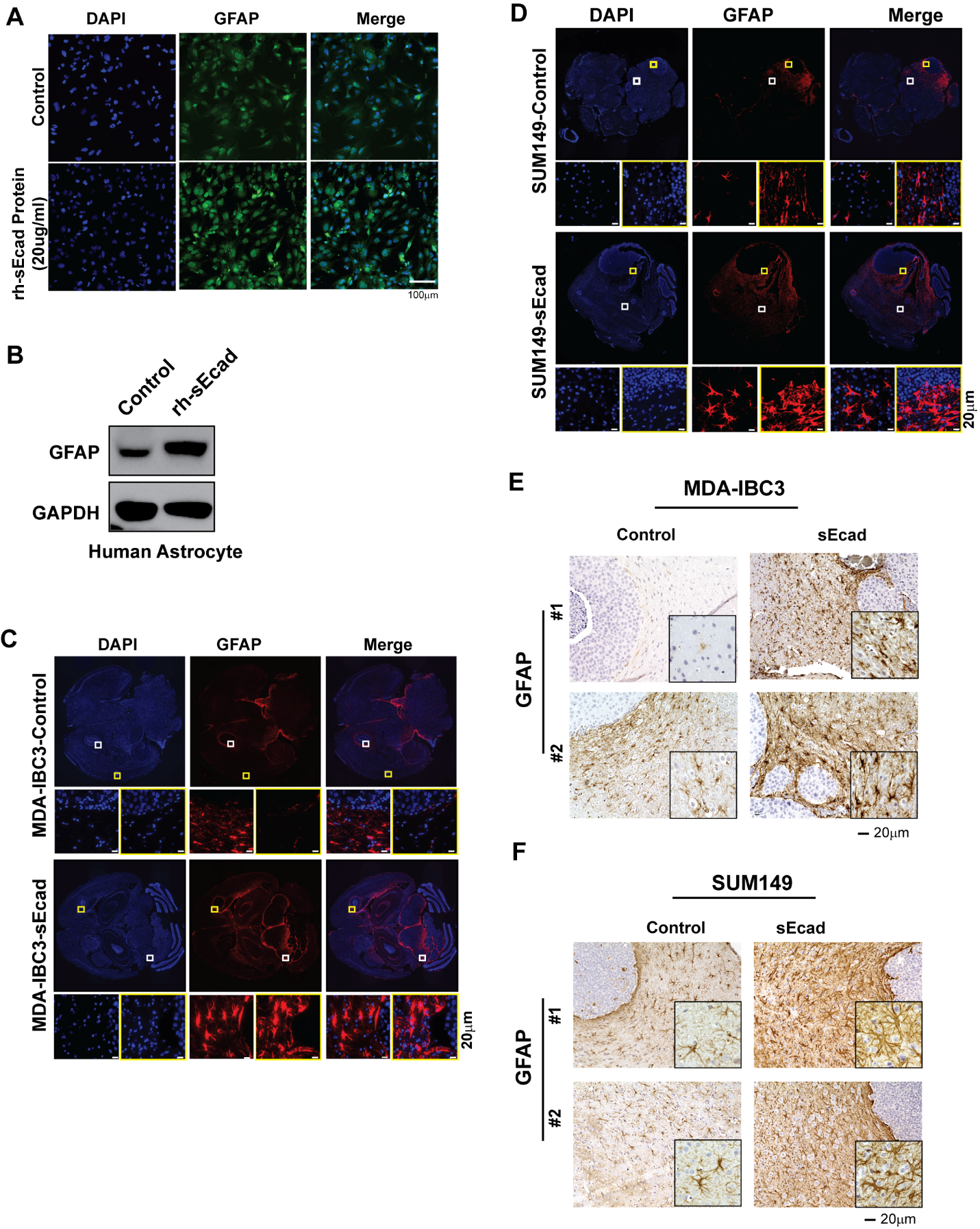


**Supplementary Figure S5. sEcad induces reactive astrocytes.**

(A, B) Astrocytes treated in vitro with sEcad recombinant protein (20 µg/mL for 36 h) show increased GFAP+ reactive astrocytes by (A) immunofluorescence (IF) staining and (B) immunoblotting; (C,D) IF staining shows that metastatic lesions from sEcad-expressing tumors have significantly higher GFAP+ reactive astrocytes vs the control group. Data shown are from mice given (C) tail-vein injections of MDA-IBC3 cells and (D) intracardiac injections of SUM-149 cells. (E, F) Immunohistochemical stains of brain metastases generated from (E) MDA-IBC3 and (F) SUM149 control and sEcad-overexpressing cells confirm the upregulated expression of GFAP in the sEcad-overexpressing cells in mouse xenograft brain metastatic lesions.


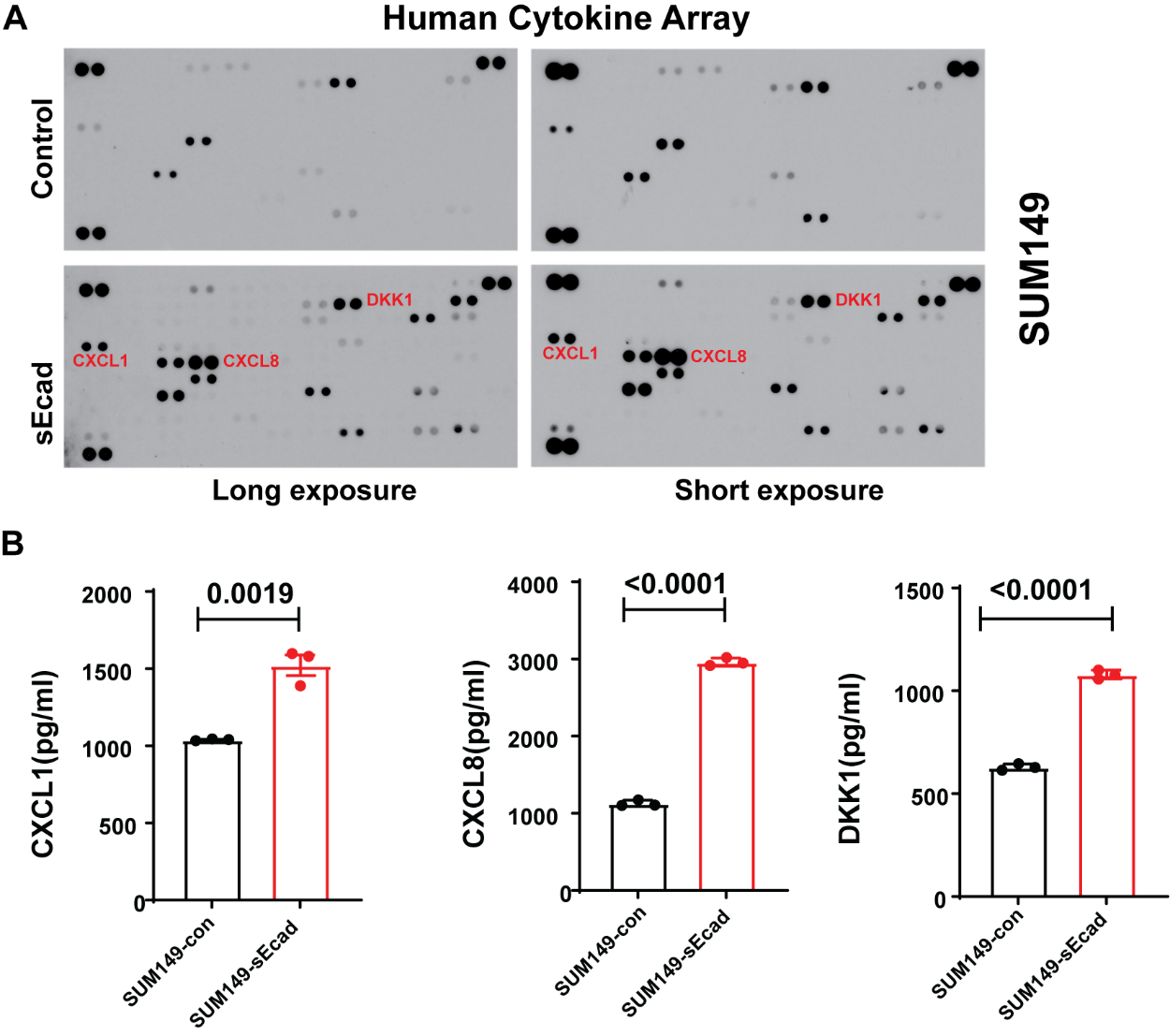


**Supplementary Figure S6. Conditioned medium from SUM149 sEcad-overexpressing cells induces CXCL1/CXCL8/DKK1 levels.** (A, B) Human cytokine array analysis of secreted factors from sEcad-overexpressing SUM149 cells. (A) Increased levels of cytokines including CXCL1, CXCL8 and DKK1 in conditioned medium from sEcad-overexpressing SUM149 cells. (B) Validation of the levels of detected cytokines from (A) by enzyme-linked immunosorbent assay.


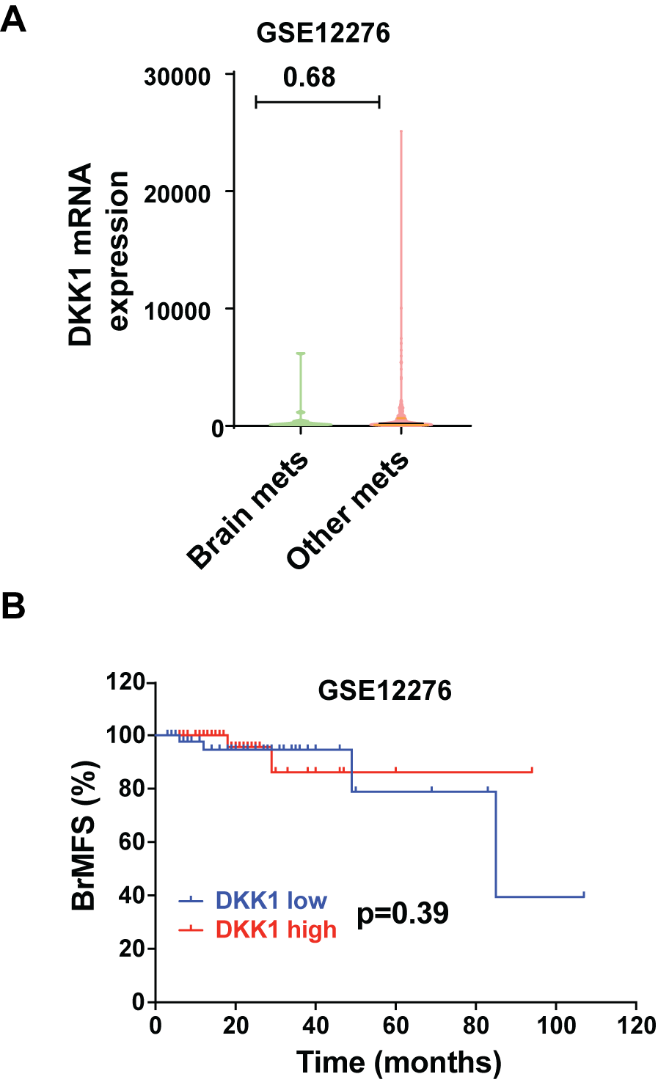


**Supplementary Figure S7. DKK1 was not correlated with brain metastasis–free survival.** (A) DKK1 expression did not differ between patients with brain metastases and patients with metastases at other sites in the Gene Expression Omnibus (GEO) database GSE12276. (B) DKK1 expression levels were not significantly correlated with brain metastasis-free survival (BrMFS), (GSE12276); low and high indicate the bottom tertile (25th) and top tertile (75th), respectively.

**
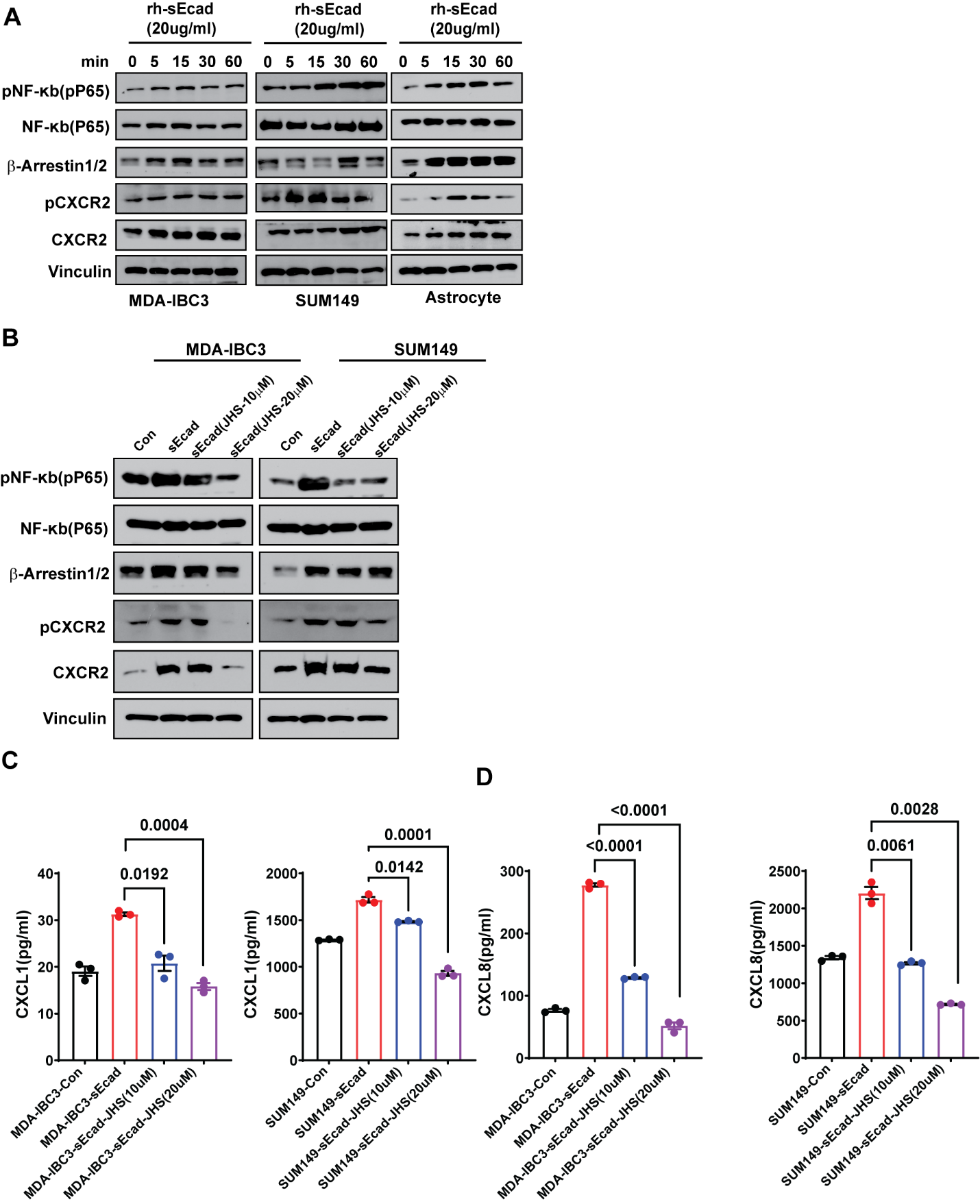
**

**Supplementary Figure S8. sEcad activates NF-κB and CXCR2 signaling in tumor cells and astrocytes, and NF-κB inhibition suppresses CXCL1/8 expression.** (A) Western blot analysis shows increased phospho-p65, β-arrestin1/2, phospho-CXCR2, and CXCR2 after treatment with recombinant sEcad (20 µg/mL) in MDA-IBC3 and SUM149 cells and in astrocytes over time. (B) sEcad-induced phospho-p65, β-arrestin1/2, and phospho-CXCR2 were reduced by the NF-κB inhibitor JSH-23 in MDA-IBC3 and SUM149 cells. (C, D) ELISA shows decreased CXCL1 and CXCL8 secretion in sEcad-expressing MDA-IBC3 and SUM149 cells after JSH-23 treatment (10 µM or 20 µM). Data are presented as mean ± SEM. P values were determined with unpaired two-tailed t tests.


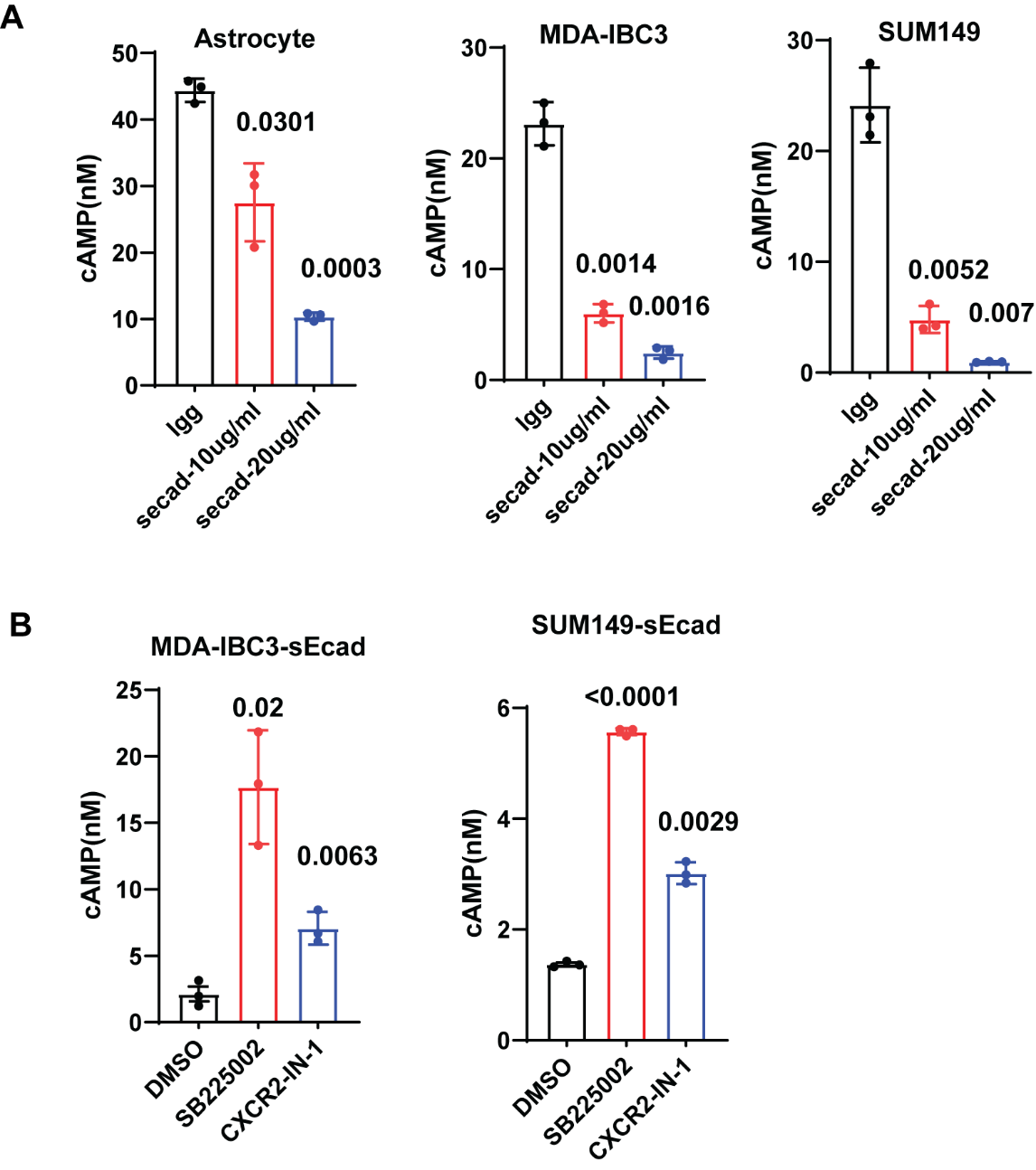


**Supplementary Figure S9. sEcad activates CXCR2 signaling in IBC cells and astrocytes.**(A) Recombinant sEcad treatment significantly reduced intracellular cAMP levels in MDA-IBC3 and SUM149 cells and in astrocytes. (B) In sEcad –overexpressing IBC3 and SUM149 cells, the CXCR2-selective antagonists SB225002 and CXCR2-IN-1 significantly increased cAMP, reversing the soluble E-cadherin–induced suppression.

**
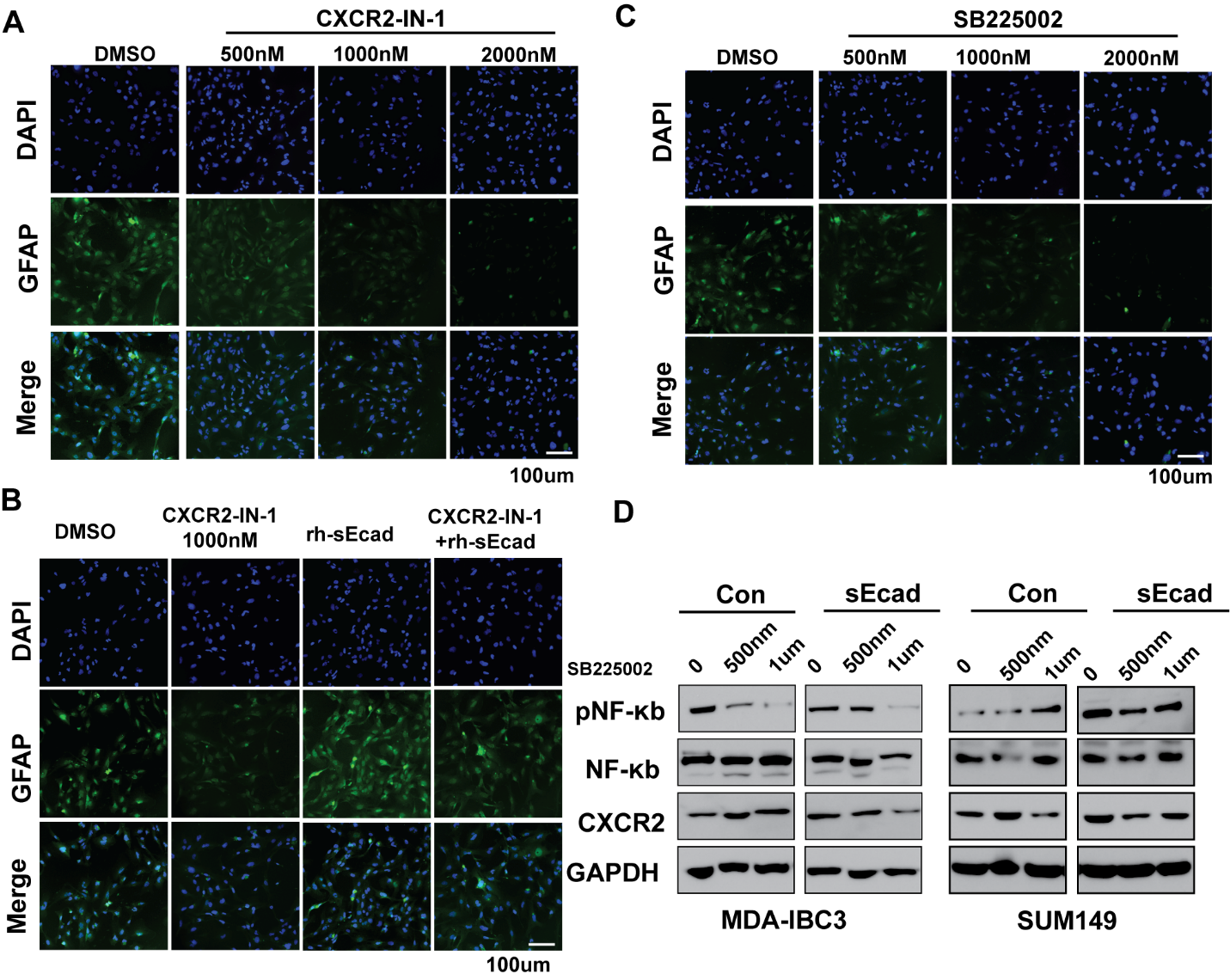
Supplementary Figure S10. Blockade of CXCR2 reduces reactive astrocytes in vitro.** (A) Immunofluorescence staining shows that CXCR2-IN-1 reduced the numbers of GFAP+ astrocytes in vitro. (B) The CXCR2 inhibitor CXCR2-IN-1 can inhibit GFAP+ astrocytes induced by recombinant sEcad protein in vitro. (C, D) The CXCR2 inhibitor SB225002 reduces GFAP and CXCR2 in human astrocytes, as shown by (C) immunofluorescence staining and (D) immunoblotting.


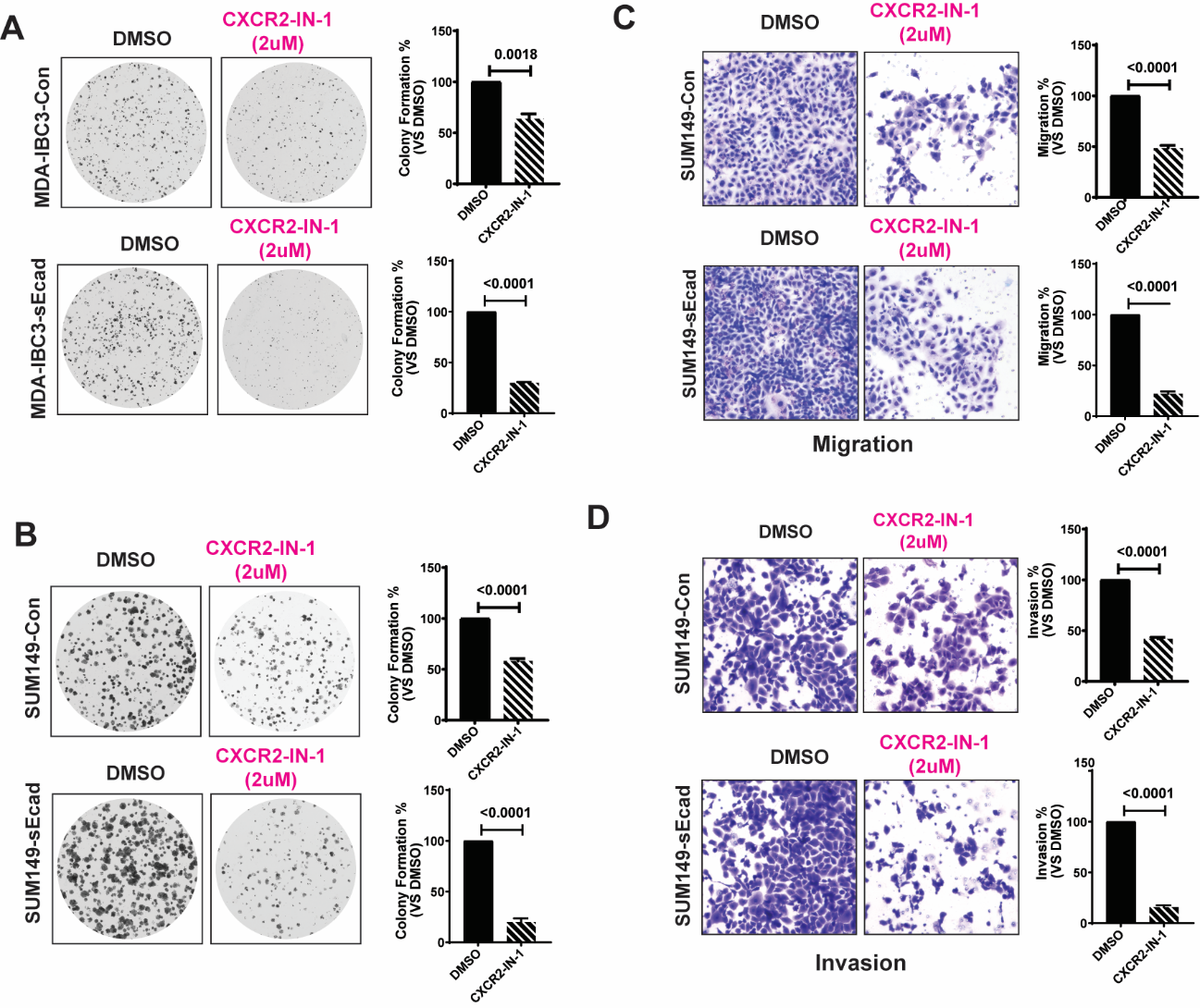


**Supplementary Figure S11. CXCR2-IN-1 inhibits oncogenic behaviors in IBC cells.** (A,B) CXCR2-IN-1 reduces colony formation in control and sEcad-overexpressing (A) MDA-IBC3 and (B) SUM149 cells. (C, D) CXCR2-IN-1 suppresses (C) migration and (D) invasion in control and sEcad-overexpressing SUM149 cells.


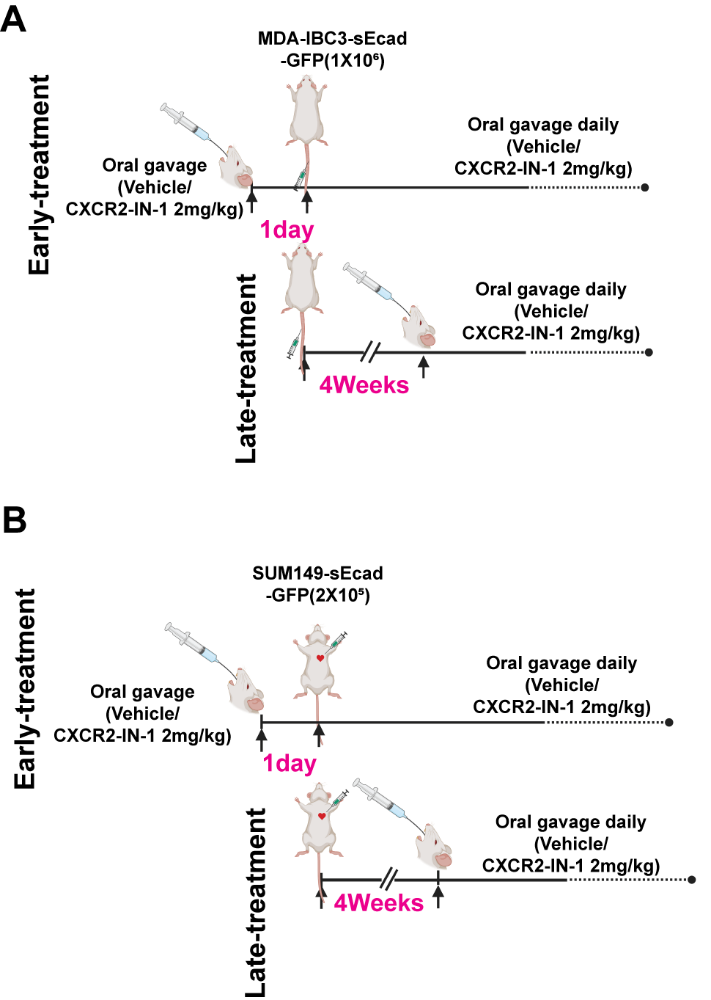


**Supplementary Figure S12.** Schematic overview of the experimental design for treating brain metastasis in MDA-IBC3 (A) and SUM149 (B) mouse models using the 'Early Treatment' or 'Late Treatment approaches.


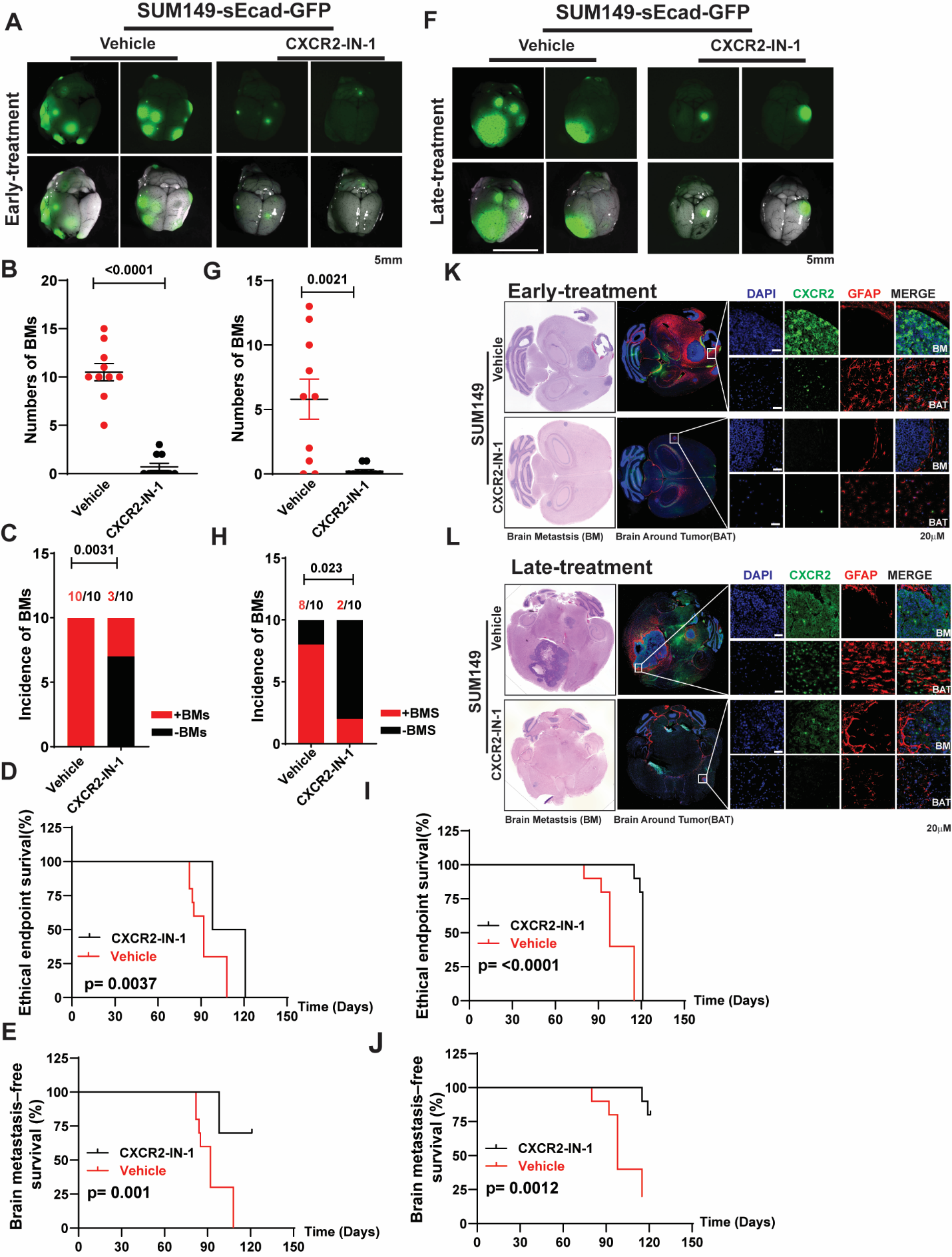


**Supplementary Figure S13. CXCR2-IN-1 reduces brain metastatic burden and improves survival in SUM149-sEcad models**. The schema for the Early and Late Treatment regimen for the SUM149 brain metastasis mouse model is shown in Figure S12B. (A-E) 'Early Treatment' group, CXCR2-IN-1 reduced brain metastases in SUM149-sEcad injected mice. (A) Representative images show reduced brain metastasis in CXCR2-IN-1-treated SUM149-sEcad mice. CXCR2-IN-1 reduced the number of brain metastasis lesions (B) and incidence of metastasis (C) and improved overall survival (D,) and brain metastasis–free survival (E). (F-J) ‘Late Treatment' group, (F) Representative images show reduced brain metastasis in CXCR2-IN-1-treated brain metastasis-bearing SUM149-sEcad (F). CXCR2-IN-1 reduced the number of brain metastasis lesions (G), incidence of metastasis (H) and improved overall survival (I) and brain metastasis–free survival (J) in mice. (K–L) H&E and immunofluorescence analyses show reduced GFAP⁺ reactive astrocytes and CXCR2 expression in both early (K) and late (L) treatment groups.


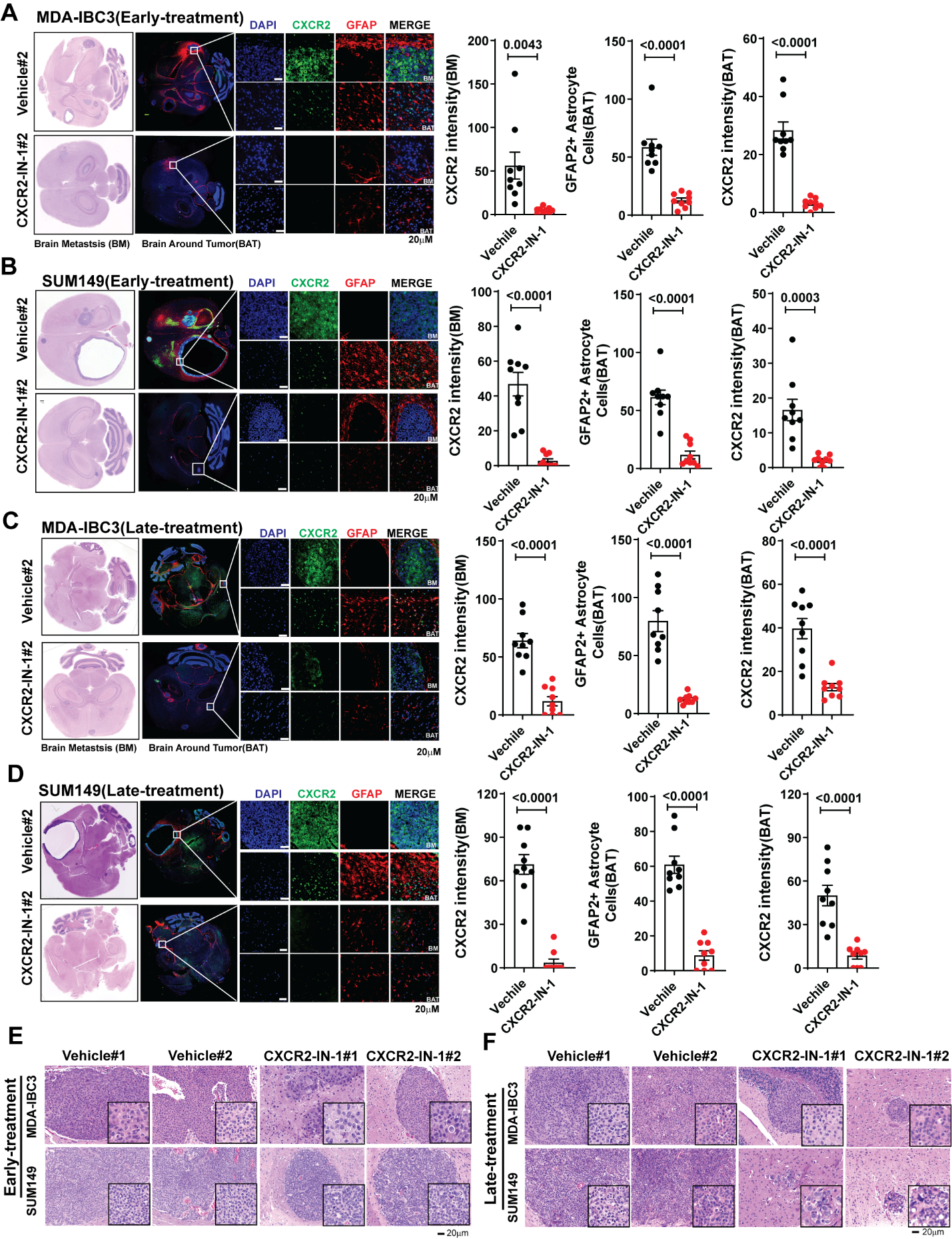


**Supplementary Figure S14. CXCR2-IN-1 reduces metastatic burden and improves survival in IBC models of brain metastasis**. (A-D) Hematoxylin and eosin and immunofluorescence stains of brain metastasis lesions in mice treated with CXCR2-IN-1 the 'Early treatment' group (A and B) and the 'Late treatment' group (C and D) indicating that CXCR2-IN-1 decreased reactive astrocytes (GFAP+) and CXCR2 expression in both MDA-IBC3-sEcad and SUM149-sEcad brain metastases models, with quantification of CXCR2- and GFAP-positive astrocytes. Data are mean ± SEM, n = 3 mice and 3 fields per mouse, with *P* values from *t* tests. (E, F) Representative hematoxylin and eosin-stained images of brain metastasis lesions generated from MDA-IBC3 and SUM149 tumors in mice treated with vehicle or CXCR2-IN-1 in the 'Early treatment' group (E) and the ‘Late treatment' groups (F).


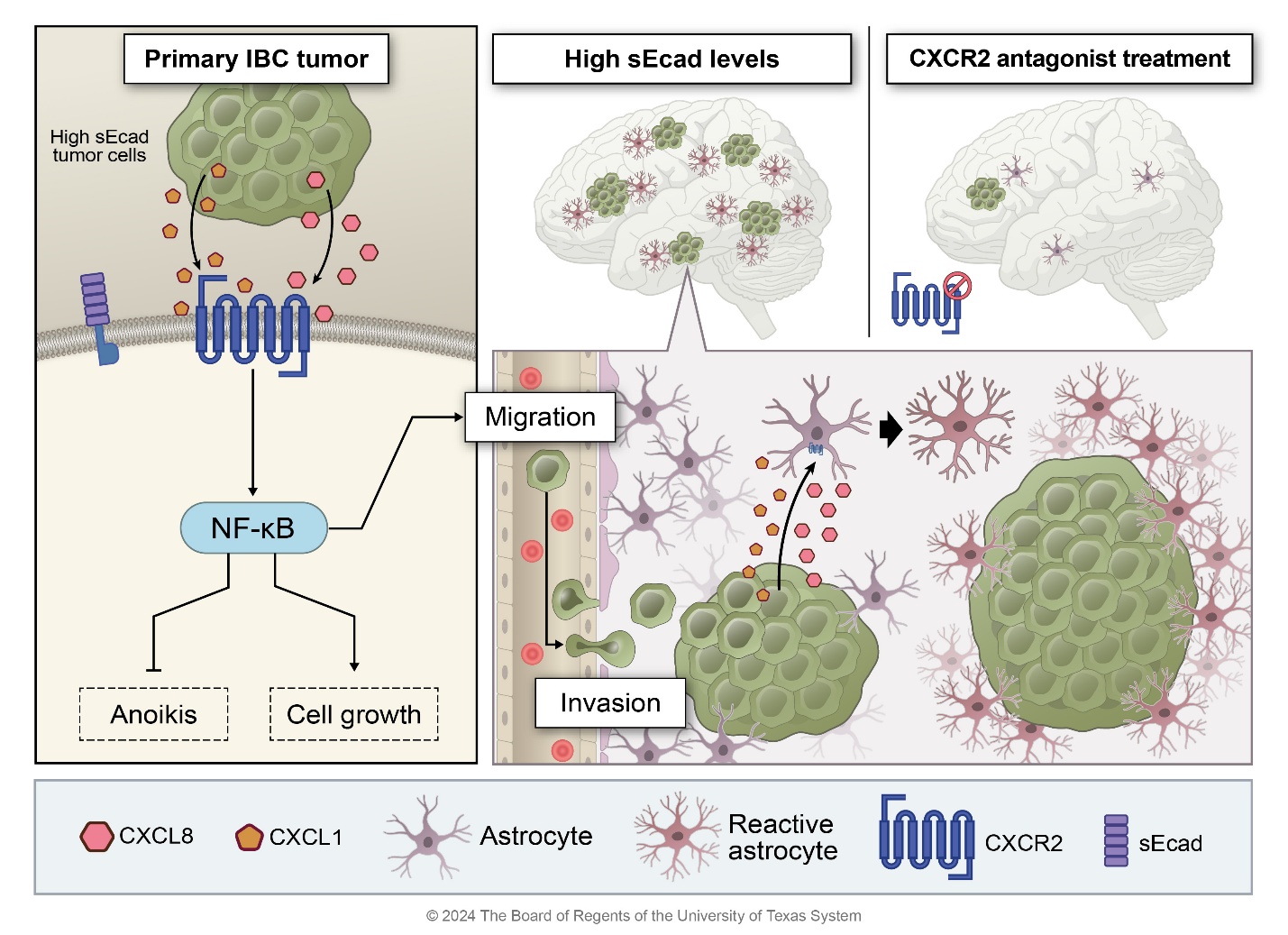
**Supplementary Figure S15. Schematic diagram.** sEcad promotes brain metastasis in IBC by activating NF-κB signaling, enhancing tumor invasion and resistance to anoikis. sEcad further modulates the brain microenvironment by activating reactive astrocytes and upregulating CXCR2 expression. Therapeutic inhibition with CXCR2-IN-1, a brain-penetrant CXCR2 antagonist, suppresses metastatic progression and prolongs survival in IBC brain metastasis models.

**Supplementary Tables S1-3**

**Supplementary Table S1. Patient characteristics**

| Covariate | Value or No. (%) |
| --- | --- |
| Patient age, years (n=304), mean±SD | 51.33±11.54 |
| sEcad, ng/mL (n=301), median (IQR) | 65.78 (52.76–80.62) |
| Race |  |
| Black | 28 (9.2%) |
| Other | 8 (2.6%) |
| Hispanic | 30 (9.9%) |
| White | 238 (78.3%) |
| Sex |  |
| Female | 304 (100%) |
| Postmenopausal |  |
| No | 135 (46.1%) |
| Yes | 158 (53.9%) |
| Unknown | 11 |
| Clinical Disease Stage at diagnosis |  |
| IIIB | 115 (37.8%) |
| IIIC | 85 (28%) |
| IV | 104 (34.2%) |
| Pathological Disease Stage |  |
| 0-1 | 63 (27.2%) |
| 2 | 27 (11.6%) |
| 3 | 92 (39.7%) |
| 4 | 50 (21.6%) |
| Unknown | 72 |
| ER status |  |
| Negative | 138 (46.3%) |
| Positive | 160 (53.7%) |
| Unknown | 6 |
| PR status |  |
| Negative | 188 (63.5%) |
| Positive | 108 (36.5%) |
| Unknown | 8 |
| HR status (either ER or PR+) |  |
| Negative | 132(44.4%) |
| Positive | 165(55.6%) |
| Unknown | 7 |
| HER2 status |  |
| Negative | 179 (61.7%) |
| Positive | 111 (38.3%) |
| Unknown | 14 |
| HR/HER2 status |  |
| HR+/HER2- | 111 (38.5%) |
| HR+/HER2+ | 50 (17.4%) |
| HR-/HER2+ | 59 (20.5%) |
| HR-/HER2- | 68 (23.6%) |
| Unknown | 16 |
| Histological Grade |  |
| I | 3 (1.1%) |
| II | 62 (22%) |
| III | 217 (77%) |
| Unknown | 22 |
| Lymphatic Invasion |  |
| No | 106 (42.7%) |
| Yes | 142 (57.3%) |
| Unknown | 56 |
| Vascular Invasion |  |
| No | 108 (43.5%) |
| Yes | 140 (56.5%) |
| Unknown | 56 |
| Response to Neoadjuvant Chemo |  |
| ADJ | 1 (0.7%) |
| Complete clinical response | 4 (2.9%) |
| Complete response | 24 (17.1%) |
| Minimal response | 17 (12.1%) |
| Progressive disease | 7 (5%) |
| Partial response | 78 (55.7%) |
| Stable disease | 9 (6.4%) |
| N/A | 164 |
| Neoadj_And_Response |  |
| No neo or no CR | 225 (90.4%) |
| CR | 24 (9.6%) |
| Neo but unknown response | 55 |
| Adjuvant Chemo |  |
| No | 253 (83.2%) |
| Yes | 51 (16.8%) |
| Neoadjuvant Radiation |  |
| No | 300 (98.7%) |
| Yes | 4 (1.3%) |
| Adjuvant Radiation |  |
| No | 141 (46.4%) |
| Yes | 163 (53.6%) |
| sEcad* |  |
| ≤95 | 260 (86.4%) |
| >95 | 41 (13.6%) |
| Missing | 3 |

*99.8 ng/mL was the 90th percentile for sEcad levels.

Abbreviations: sEcad, soluble E-cadherin; ER, estrogen receptor; PR, progesterone receptor; Chemo, chemotherapy; ADJ, adjuvant; N/A, not applicable; CR, complete response; Neo, neoadjuvant (pre-operative) chemotherapy.

**Supplementary Table S2. Univariate Cox regression analysis for overall and breast-cancer–specific deaths**

| Overall Survival* BC-Specific Survival**  BC-Specific Survival (120 BC deaths) | | | | | |
| --- | --- | --- | --- | --- | --- |
| **Covariate** | **HR (95% CI)** | ***P* Value** |  | **HR (95% CI)** | ***P* Value** |
| Age |  |  |  |  |  |
| ≤50 years | 1.000 |  |  | 1.000 |  |
| >50 years | 0.997 (0.703-1.415) | 0.9883 |  | 0.895 (0.625-1.281) | 0.5441 |
| Race |  |  |  |  |  |
| Not Black | 1.000 |  |  | 1.000 |  |
| Black | 1.793 (1.075-2.991) | 0.0253 |  | 1.921 (1.149-3.212) | 0.0128 |
| Postmenopausal |  |  |  |  |  |
| No | 1.000 |  |  | 1.000 |  |
| Yes | 1.021 (0.718-1.453) | 0.9068 |  | 0.980 (0.683-1.408) | 0.9149 |
| Clinical Disease Stage |  |  |  |  |  |
| IIIB | 1.000 |  |  | 1.000 |  |
| IIIC | 1.947 (1.225-3.092) | 0.0048 |  | 1.940 (1.193-3.156) | 0.0076 |
| IV | 2.406 (1.552-3.730) | <.0001 |  | 2.661 (1.693-4.184) | <0.0001 |
| Pathological Stage |  |  |  |  |  |
| 0-1 | 1.000 |  |  | 1.000 |  |
| 2 | 1.976 (0.818-4.773) | 0.1300 |  | 2.145 (0.827-5.563) | 0.1167 |
| 3 | 3.097 (1.590-6.031) | 0.0009 |  | 3.430 (1.654-7.116) | 0.0009 |
| 4 | 2.769 (1.350-5.682) | 0.0055 |  | 3.386 (1.566-7.319) | 0.0019 |
| Estrogen Receptor Status |  |  |  |  |  |
| Negative | 1.000 |  |  | 1.000 |  |
| Positive | 0.673 (0.473-0.958) | 0.0280 |  | 0.656 (0.456-0.943) | 0.0230 |
| Progesterone Receptor Status |  |  |  |  |  |
| Negative | 1.000 |  |  | 1.000 |  |
| Positive | 0.456 (0.302-0.689) | 0.0002 |  | 0.471 (0.309-0.718) | 0.0005 |
| Hormone Receptor Status |  |  |  |  |  |
| Negative | 1.000 |  |  | 1.000 |  |
| Positive | 0.683 (0.480-0.973) | 0.0348 |  | 0.669 (0.465-0.963) | 0.0305 |
| HER2 Status |  |  |  |  |  |
| Negative | 1.000 |  |  | 1.000 |  |
| Positive | 0.277 (0.174-0.441) | <0.0001 |  | 0.281 (0.174-0.453) | <0.0001 |
| HR/HER2 Status |  |  |  |  |  |
| HR+/HER2– | 1.000 |  |  | 1.000 |  |
| HR+/HER2+ | 0.392 (0.198-0.775) | 0.0071 |  | 0.376 (0.183-0.770) | 0.0075 |
| HR–/HER2+ | 0.383 (0.203-0.723) | 0.0031 |  | 0.407 (0.214-0.772) | 0.0059 |
| HR–/HER2– | 2.137 (1.427-3.200) | 0.0002 |  | 2.148 (1.416-3.260) | 0.0003 |
| Grade |  |  |  |  |  |
| I-II | 1.000 |  |  | 1.000 |  |
| III | 1.089 (0.700-1.695) | 0.7058 |  | 1.124 (0.709-1.780) | 0.6194 |
| Lymphatic Invasion |  |  |  |  |  |
| Negative | 1.000 |  |  | 1.000 |  |
| Positive | 1.945 (1.266-2.989) | 0.0024 |  | 2.152 (1.367-3.388) | 0.0009 |
| Vascular Invasion |  |  |  |  |  |
| Negative | 1.000 |  |  | 1.000 |  |
| Positive | 2.027 (1.319-3.112) | 0.0013 |  | 2.241 (1.424-3.528) | 0.0005 |
| Response to Neoadjuvant Chemo |  |  |  |  |  |
| No | 1.000 |  |  | 1.000 |  |
| Res | 0.190 (0.060-0.599) | 0.0046 |  | 0.131 (0.032-0.532) | 0.0045 |
| Receipt of Adjuvant Chemo |  |  |  |  |  |
| No | 1.000 |  |  | 1.000 |  |
| Yes | 0.451 (0.262-0.779) | 0.0043 |  | 0.442 (0.251-0.776) | 0.0045 |
| Receipt of Neoadjuvant Radiation |  |  |  |  |  |
| No | 1.000 |  |  | 1.000 |  |
| Yes | 1.770 (0.561-5.587) | 0.3305 |  | 1.855 (0.587-5.864) | 0.2925 |
| Receipt of Adjuvant Radiation |  |  |  |  |  |
| No | 1.000 |  |  | 1.000 |  |
| Yes | 0.368 (0.257-0.525) | <0.0001 |  | 0.339 (0.234-0.491) | <0.0001 |
| sEcad |  |  |  |  |  |
| ≤95 | 1.000 |  |  | 1.000 |  |
| >95 | 1.596 (1.007-2.529) | 0.0466 |  | 1.614 (1.007-2.587) | 0.0466 |

*127 deaths

**120 deaths

Abbreviations: HR, hazard ratio; CI, confidence interval; Chemo, chemotherapy sEcad, soluble E-cadherin.

**Supplementary Table S3.** Multivariate Cox regression analysis of overall survival and breast cancer–specific survival among patients with IBC

|  |  | Overall survival | | Breast cancer–specific survival | |
| --- | --- | --- | --- | --- | --- |
|  |  | Multivariate Cox Model 1 (N=285) | | Multivariate Cox Model 1 (n=278) | |
| **Covariate** | **Level** | **HR (95% CI)** | ***P* Value**   \| **HR (95% CI)** \| **P-value** \| \| --- \| --- \| \| **1.000** \|  \| \| **2.490 (1.465-4.231)** \| **0.0008** \| \| **3.363 (2.073-5.453)** \| **<.0001** \| \| **1.000** \|  \| \| **0.387 (0.188-0.798)** \| **0.0101** \| \| **0.343 (0.179-0.655)** \| **0.0012** \| \| **2.318 (1.514-3.549)** \| **0.0001** \| \| **1.000** \|  \| \| **1.898 (1.153-3.123)** \| **0.0117** \| | **HR (95% CI)** | ***P* Value** |
| sEcad | ≤95 | 1.000 |  | 1.000 |  |
|  | >95 | 1.905 (1.174-3.093) | 0.0091 | 1.898 (1.153-3.123) | 0.0117 |
| Clin_stage_side1 | Iiib | 1.000 |  | 1.000 |  |
|  | Iiic | 2.489 (1.508-4.108) | 0.0004 | 2.490 (1.465-4.231) | 0.0008 |
|  | Iv | 2.981 (1.871-4.748) | <.0001 | 3.363 (2.073-5.453) | <.0001 |
| HR/HER2 | HR+/HER2- | 1.000 |  | 1.000 |  |
|  | HR+/HER2+ | 0.402 (0.202-0.801) | 0.0095 | 0.387 (0.188-0.798) | 0.0101 |
|  | HR-/HER2+ | 0.321 (0.169-0.611) | 0.0005 | 0.343 (0.179-0.655) | 0.0012 |
|  | HR–/HER2- | 2.291 (1.518-3.459) | <.0001 | 2.318 (1.514-3.549) | 0.0001 |
