# Supplementary material for: Soluble E-cadherin-CXCL1-CXCR2 axis as a therapeutic vulnerability in inflammatory breast cancer brain metastasis
Source: Neuro Oncol. 2026 Jan 23;28(5):1220–36. doi: 10.1093/neuonc/noag012 (PMC13158929; doi:10.1093/neuonc/noag012)
Supplement: noag012_Supplementary_Data [file noag012_supplementary_data.zip › SI Methods.docx]

**SUPPLEMENTARY METHODS**

**Soluble E-cadherin–CXCL1–CXCR2 Axis as a Therapeutic Vulnerability in Inflammatory Breast Cancer Brain Metastasis**

**Xiaoding Hu,****^1,7^ Yun Xiong,^2^ Emilly S Villodre,^1,7^ Huimin Zhang,^2^ Isabella R Longa^1,7^, Juhee Song,^3^ Natalie Fowlkes,^4^ Savitri Krishnamurthy,^5,7^ Marissa Rylander,^8^ Chandra Bartholomeusz,**^1,7^ **Debu Tripathy,^1 7^ Wendy A Woodward,^6,7^ Junjie Chen,^2^ and Bisrat G Debeb^1,7,*^**

Departments of ^1^Breast Medical Oncology, ^2^Experimental Radiation Oncology, ^3^Biostatistics ,^4^ Veterinary Medicine and Surgery, ^5^Pathology, ^6^Breast Radiation Oncology, and ^7^MD Anderson Morgan Welch Inflammatory Breast Cancer Clinic and Research Program, The University of Texas MD Anderson Cancer Center, Houston TX.^8^Department of Biomedical Engineering, The University of Texas at Austin, Austin, TX

**Enzyme-linked immunosorbent assay of IBC samples**

Serum samples were identified from a prospectively maintained inflammatory breast cancer (IBC) registry and biobank. All patients provided informed consent for collection and use of the samples on an institutional review board (IRB)-approved protocol. Levels of soluble E-cadherin in serum from 348 patients with IBC were analyzed by enzyme-linked immunosorbent assay (ELISA) (R&D Systems, Minneapolis, MN, USA #DCADE0) according to the manufacturer’s instructions. Samples were assayed in duplicate. For conditioned-medium ELISA, cancer cells were cultured with serum-free medium for 36 h, after which the conditioned medium was collected and subjected to ELISA for CXCL1, CXCL8 and DKK1 (R&D Systems, Minneapolis, MN, USA #DGR00B, #D8000C, and #DKK100B, respectively).

**Cell cultures**

SUM149 and SUM190 cell lines was purchased from Asterand (Detroit, MI), and the MDA-IBC3 cell line was generated in the laboratory of Dr. Wendy Woodward at MD Anderson.^1, 2^ The human triple-negative breast cancer (TNBC) cell line BCX010, derived from a patient with triple-negative inflammatory breast cancer, was generously donated by Dr. Funda Meric-Bernstam (MD Anderson Cancer Center).^3^ The cell culture conditions are described elsewhere.^4, 5^ HEK293T cells were obtained from the American Type Culture Collection (ATCC; Manassas, VA, USA) and were cultured in Dulbecco’s modified Eagle’s medium (DMEM) supplemented with 10% fetal bovine serum (FBS) and 1% penicillin and streptomycin (Pen/Strepto; #15140122, Invitrogen, Carlsbad, CA, USA) at 37°C in a humidified incubator with 5% CO_2_. Normal human astrocytes were cultured with High DMEM (HyClone #SH30243.01 GE), 10% FBS (Gibco #16000-044), 1% Pen/Strepto (#15-140-122, Fisher Scientific), 5 μg/mL insulin (Cell Applications #128-100), 10 μM hydrocortisone (#H0888, Sigma), and 5 μg/mL N-acetylcysteine (A9165, Sigma). The human microvascular endothelial cell line HBEC-5i (CRL-3245), mouse astrocyte line C8-D1A (CRL-2541), human umbilical vein endothelial cells (HUVECs) (PCS-100-010), and mouse endothelial cells bEnd.3 (CRL-2299) cells were acquired from the ATCC (Manassas, VA, USA) and cultured per the recommended protocols. Bend3 endothelial cells and C8-D1A astrocytes were cultured in ATCC-formulated DMEM (30-2002, ATCC) containing 10% FBS (30-2020, ATCC). HMC3 cells were cultured in Eagle's minimum essential medium (EMEM) (30-2003, ATCC) basal medium containing 10% FBS (30-2020, ATCC). HUVECs were cultured with Vascular Cell Basal Medium (PCS-100-030, ATCC), Endothelial Cell Growth Kit-VEGF (PCS-100-041, ATCC), and Penicillin-Streptomycin-Amphotericin B Solution (PCS-999-002, ATCC).

To collect conditioned medium, cancer cells were seeded into culture dishes and incubated for 24 hours. After, cells were washed twice with 1X phosphate-buffered saline (PBS), 10 mL of fresh medium without FBS was added, the cells incubated for another 36 hours at 37°C, and then the medium was collected and filtered with a 0.2-μm syringe filter. For cytokine assays, the amounts used are described in the manufacturer’s protocol. For astrocyte co-culture experiments, the cells were washed twice with 1X PBS after 24 hours of attachment and then treated with 30% conditioned medium and 70% culture medium for 24 hours to collect samples for western blot assays.

**Plasmid construction and generation of stable cells**

Soluble E-cadherin cDNA (1-707aa) was amplified from E-cadherin (Gene ID: 999) cDNA by using the following primers (Forward primer: ATGGGCCCTTGGAGCCGCAGC; Reverse primer: AATTTGCAATCCTGCTTCGAC) and then cloned into modified LentiV_Blast-Flag vector (#111887, Addgene). Stable Flag-tagged sEcad-overexpressing IBC cell lines (MDA-IBC3, SUM190, SUM149, and BCX010) were established.

**Antibodies and reagents**

Anti-GAPDH (#8884), Anti- β-Actin (#4967), Anti-Tubulin (#2148S), Anti-HA-Tag (#C29F4) ([3724S)](http://www.abcam.com/ha-tag-antibody-hac5-ab18181.html), Anti-NF-κB p65 (#8242), Anti-Phospho-NF-κB p65 (Ser536) (#3033), Anti-Caspase-3 (#9662), Anti-Caspase7 (#9494), Anti-Cleaved Caspase-9 (Asp353) (#9509), Anti-glial fibrillary acidic protein (GFAP) (#3670S), Anti-Ki67 (#12202), Anti-p44/42 (Erk1/2) (#4696), Anti-Phospho-p44/42 (Erk1/2) (Thr202/Tyr204) (#4370), Anti-Phospho-IkappaB alpha (Ser32) (#2859), Anti-IkappaB alpha (44D4) (#4812) and Anti-beta-Arrestin 1/2 (D24H9) (#4674) antibodies were purchased from Cell Signaling Technology (Beverly, MA, USA). All of the secondary antibodies [Anti-mouse-HRP (#7076S), Anti-rabbit-HRP (#7074S), Anti-mouse-Alexa Fluor 555 (#4409S), Anti-mouse-Alexa Fluor 488 (#4408S), Anti-rabbit-Alexa Fluor 594 (#8889S), and Anti-rabbit-Alexa Fluor 488 (#4412S)] were also purchased from Cell Signaling Technology. Anti-CXCR2 (ab65968) and Anti-CXCR2 (phospho S347) (ab61100) antibodies were purchased from Abcam (Cambridge, MA, USA).  [Human E-Cadherin Antibody (MAB1838), Recombinant Human E-Cadherin Protein-CF (sEcad protein) (8505-EC), Human E-Cadherin Quantikine ELISA Kit ( #DCADE0B), Human CXCL1/GRO alpha Quantikine ELISA Kit (#DGR00B), Human IL-8/CXCL8 Quantikine ELISA Kit (#D8000C), Human Dkk-1 Quantikine ELISA Kit (#DKK100B), and Proteome Profiler Human Cytokine Array Kit (#ARY005B) was purchased from R&D system (USA). Anti-Flag (#F1804), RIPA buffer (#R0278), and the sEcad-neutralizing antibody DECMA1 (#MABT26) were purchased from Sigma (St Louis, MO, USA). SB225002 (#S7651) was purchased from Selleck (Houston, TX, USA). CXCR2-IN-1 was purchased from MedChem Express (#HY-101022).](https://www.rndsystems.com/products/recombinant-human-decorin-protein-cf_143-de)

**Western blotting**

Total protein was extracted from cancer cells by using radioimmunoprecipitation assay (RIPA) buffer (Sigma) with 10 µL/mL phosphatase and 10 µL/mL protease inhibitor cocktail. Forty micrograms of protein lysate from each sample were electrophoretically separated with a 10% SDS–PAGE gel and then transferred to a polyvinylidene difluoride membrane. SDS-PAGE and immunoblotting were done as previously described by us.^4, 6^ Membranes were incubated with the corresponding primary antibodies overnight at 4°C and then incubated with secondary antibodies (1:5000) anti-rat IgG (#HAF005, R&D Systems) and anti-rabbit IgG (#7074, Cell Signaling) for 2 h at room temperature. GAPDH/B-actin/Vinculin was used as an internal control.

**Lentiviral production and transduction**

Protocol details are described elsewhere.^4, 6^ Briefly, Lipofectamine 3000 (Invitrogen, USA) DNA mixture (10 µg LentiV_Blast-Flag-sEcadherin/Luc-GFP, 7.5 µg of psPAX2 packaging plasmid and 2.5 µg of pMD2.G enveloping plasmid) were incubated overnight with HEK293T cells. The culture medium was then removed and replaced with fresh medium. The supernatant containing the virus was collected, filtered through a 0.45-μm HV Durapore membrane (EMD Millipore) to remove cells and large debris, and concentrated by ultracentrifugation. Target cells with a confluence of about 70% were used for transduction. The medium was changed 2 hours before transduction. Lentiviral transduction was performed in the presence of 8 μg/mL polybrene (Sigma-Aldrich, USA) for 24 hours, followed by replacement with fresh medium.

**Soft agar / anchorage-independent growth assay**

Cell growth in soft agar for anchorage-independent growth was assessed as described elsewhere.^6, 7^ Briefly, 1 mL of complete medium containing 1% agarose was added evenly to each well of a 12-well plate, while ensuring no bubbles were present, until it completely solidified (bottom layer). MDA-IBC3 cells or SUM190 (5000 cells each) or SUM149 cells or BC0X10 cells (8000 cells each) were suspended in 0.5% agarose in complete medium in the presence or absence of sEcad recombinant protein (20 μg/mL) and DECMA1 (20 μg/mL) (top layer). Then, the top layer mixture of cells and agarose was layered onto the solidified bottom layer and the layers were incubates at 37°C for 3 weeks. Colonies were evaluated with an inverted microscope (Nikon, Tokyo, Japan), and 10 random areas were chosen to observe and photograph. In addition, colonies were stained with MTT, and colonies >80 µm in diameter were counted with the GelCount system (Oxford Optronix Ltd). The same experiments and analyses were done with control and sEcad-overexpressing MDA-IBC3, SUM190, SUM149, or BCX010 cells.

**Immunohistochemical and i****mmunofluorescence staining**

Details of the immunofluorescence staining protocol for cultured cells was described by us previously.^4^ Astrocytes were grown on Millicell EZ SLIDE 4-well glass (PEZGS0416) and treated with the corresponding proteins or inhibitors. Cells were then stained with the primary antibodies anti-GFAP (1:200 dilution, CST, #3670S) and anti-CXCR2 (1:100 dilution, Abcam, #ab65968) for 2 hours at room temperature. Staining was visualized by using Alexa-Fluor 594-conjugated secondary antibody (diluted 1:500) and Alexa-Fluor 468-conjugated secondary antibody (diluted 1:500) and then stained with 4'6-diamidino-2-phenylindole (DAPI) with indole (0.5 μg/mL) for 15 minutes to label the nuclei. Finally, the coverslips were washed five times with 1X PBS. Immunofluorescence microscopy images were obtained with a Keyence BZ-X800 microscope (Keyence Corporation of America).

Formalin-fixed, paraffin-embedded sections of brain tissues were stained with hematoxylin and eosin (H&E) at MD Anderson’s Pathology core facility with standard and validated protocols. Slides were analyzed by a pathologist specializing in breast cancer (SK). Immunohistochemical staining was done with biotinylated secondary antibodies (Vector), biotin-avidin-peroxidase complex (Vector), and diaminobenzidine (brown; Sigma) or Vector blue (Vector) as the developing agents. Fluorescence immunohistochemical staining was done with the Alexa Fluor-tagged secondary antibodies Alexa 488 (green) or Alexa 594 (red). Primary antibodies used were rabbit anti–CXCR2 (1:100), mouse anti-GFAP (1:500), and DAPI (Molecular Probes, D-1306) was used as a fluorescent counterstain. Stained sections were examined and photographed with bright-field and fluorescence microscopy with a Keyence BZ-X800 microscope (Keyence Corporation of America).

**Endothelial cell adhesion assay**

Human brain microvascular endothelial HBEC-5i cells were seeded into 6-well plates and allowed to form a confluent monolayer for 24 hours. The tops of the monolayers were then seeded with sEcad-overexpressing or control MDA-IBC3 / SUM149 cells labeled with green fluorescent protein (GFP). The cells were allowed to incubate for 30 minutes, medium was aspirated, and cells were washed with PBS twice to remove nonadherent cells. The fluorescent tumor cells were imaged, and the numbers of cells were counted per field as described previously.^8^

**In vitro trans–blood brain barrier migration assay**

Mouse astrocytes C8-D1A (5× 10^5^) were plated on the bottom side of a transwell, and cell medium was refreshed every 15 mins for 6 hours. The transwell was then inverted back and 2.5× 10^5^ bEnd.3 cells were plated on the top side of the membrane, after which the transwell was incubated at 37°C for 3 days to allow formation of blood-brain barrier (BBB). Transwells with intact BBB were washed twice with PBS to remove the serum and inserted into 24-well plates containing the tumor-conditioned medium from the tumor cells in the bottom chamber after the cells had been starved for 24 hours. GFP-labeled tumor cells were then plated onto the top chamber and the migratory ability of the tumor cells was noted 20 hours later.^8^

***In Vitro* angiogenesis assay**

About 2× 10^4^ HUVECs were plated on Extracellular Matrix Gel provided in an *in vitro* angiogenesis kit (Abcam, ab204726) with tumor-conditioned medium and monitored for tube formation up to 18 hours. For treatment, 20 μg/mL sEcad recombinant protein, 20 μg/mL DECMA1 neutralizing antibody, and IgG control were added to the fresh HUVEC medium for 24 hours. Branch points were quantified per microscopic field.

**cAMP assay and analysis**cAMP levels were measured with a cAMP Assay Kit (Abcam, ab234585) according to the manufacturer’s instructions. Recombinant sEcad protein (20 µg/mL) was used to treat MDA-IBC3 and SUM149 cells and astrocytes. For inhibitor studies, sEcad-overexpressing MDA-IBC3 and SUM149 cells were treated with CXCR2-IN-1 (2 µM) or SB225002 (2 µM).

**In vitro migration and invasion assays**

Protocol details are described elsewhere.^4^ Migration assays were done with 24-well transwell plates (Corning, Inc. USA). For invasion assays, the upper chamber was pre-coated with Matrigel (BD Biosciences, Franklin Lakes, NJ, USA). To investigate the effects of sEcad overexpression on IBC cell migration and invasion, 5 × 10^4^ Control or sEcad-overexpressing SUM149 or BCX010 cells were placed in 100 μL of serum-free medium and seeded into the upper chamber with or without Matrigel. IgG, 20 μg/mL sEcad-recombinant protein, or 20 μg/mL DECMA1 neutralizing antibody were added to the upper chamber containing 5 × 10^4^ SUM149 or BCX010 cells, and migration and invasion were analyzed as follows. After 24 hours of culture, cells that had migrated to the bottom surface were fixed in 4% paraformaldehyde (#AAJ19943K2, Thermo Scientific, USA) for 30 minutes and stained with 1% crystal violet solution for 25 minutes, and the non-migrated cells were gently removed from the upper chamber with a cotton swab. Under a microscope (Nikon eclipse Ti camera, NY, USA), ten randomly chosen visual fields were recorded and analyzed with ImageJ software.

**REFERENCES**

1. Klopp AH, Lacerda L, Gupta A, et al. Mesenchymal Stem Cells Promote Mammosphere Formation and Decrease E-Cadherin in Normal and Malignant Breast Cells. Plos One 2010;5.

2. Debeb BG, Lacerda L, Anfossi S, et al. miR-141-Mediated Regulation of Brain Metastasis From Breast Cancer. Jnci-J Natl Cancer I 2016;108.

3. McAuliffe PF, Evans KW, Akcakanat A, et al. Ability to Generate Patient-Derived Breast Cancer Xenografts Is Enhanced in Chemoresistant Disease and Predicts Poor Patient Outcomes. Plos One 2015;10:e0136851.

4. Hu XD, Villodre ES, Larson R, et al. Decorin-mediated suppression of tumorigenesis, invasion, and metastasis in inflammatory breast cancer. Commun Biol 2021;4.

5. Villodre ES, Hu XD, Eckhardt BL, et al. NDRG1 in Aggressive Breast Cancer Progression and Brain Metastasis. Jnci-J Natl Cancer I 2022;114:579-591.

6. Hu X, Zhao Y, Wei L, et al. CCDC178 promotes hepatocellular carcinoma metastasis through modulation of anoikis. Oncogene 2017;36:4047-4059.

7. Du F, Zhao XD, Fan DM. Soft Agar Colony Formation Assay as a Hallmark of Carcinogenesis. Bio-Protocol 2017;7.

8. Chang GQ, Shi L, Ye YQ, et al. YTHDF3 Induces the Translation of m^6^ A-Enriched Gene Transcripts to Promote Breast Cancer Brain Metastasis. Cancer Cell 2020;38:857-+.
